# Recent advances in proteomics and metabolomics in plants

**DOI:** 10.1186/s43897-022-00038-9

**Published:** 2022-07-23

**Authors:** Shijuan Yan, Ruchika Bhawal, Zhibin Yin, Theodore W. Thannhauser, Sheng Zhang

**Affiliations:** 1 Guangdong Key Laboratory for Crop Germplasm Resources Preservation and Utilization, Agro-biological Gene Research Center, Guangdong Academy of Agricultural Sciences, Guangzhou, China; 2 Proteomics and Metabolomics Facility, Institute of Biotechnology, Cornell University, 139 Biotechnology Building, 526 Campus Road, Ithaca, NY 14853 USA; 3 R. W. Holley Center for Agriculture & Health, USDA/ARS, Ithaca, NY USA

**Keywords:** LC-MS, GC-MS, Single cell proteomics, Phosphoproteomics, Tandem mass tag, Fluxomics, Spatially resolved metabolomics, Mass spectrometry imaging

## Abstract

Over the past decade, systems biology and plant-omics have increasingly become the main stream in plant biology research. New developments in mass spectrometry and bioinformatics tools, and methodological schema to integrate multi-omics data have leveraged recent advances in proteomics and metabolomics. These progresses are driving a rapid evolution in the field of plant research, greatly facilitating our understanding of the mechanistic aspects of plant metabolisms and the interactions of plants with their external environment. Here, we review the recent progresses in MS-based proteomics and metabolomics tools and workflows with a special focus on their applications to plant biology research using several case studies related to mechanistic understanding of stress response, gene/protein function characterization, metabolic and signaling pathways exploration, and natural product discovery. We also present a projection concerning future perspectives in MS-based proteomics and metabolomics development including their applications to and challenges for system biology. This review is intended to provide readers with an overview of how advanced MS technology, and integrated application of proteomics and metabolomics can be used to advance plant system biology research.

## Introduction

Plant research includes a wide range of scientific disciplines that involves all aspects of plant biology. It’s importance is becoming more apparent as we recognize how broadly plants impact human life, through nutrition, food security, medicine, biofuels and environmental sustainability (Gemperline et al. 2016a). Plant research is essential to address key issues in environmental science, agriculture and medicine that are closely associated with human health and wellbeing. Over the past decade, systems biology studies have increasingly become the main stream in plant research (Sheth and Thaker 2014), leveraging the development of omics technologies through multi-omics integration and data processing (Feussner and Polle 2015; Ramalingam et al. 2015; Pazhamala et al. 2021).

The proteome is a time-dependent expression of an organism’s genome that is characterized with regard to protein localization, interactions, modification and turnover. Proteomics is the systematic identification and quantification of an organism’s proteome at a given time. It is a useful approach to discover biomarkers for specific stimuli, or for determining relevant biological pathways, molecular mechanisms and functional networks at the levels of biological organization (i.e., cell, tissue, organ etc.). Over the last two decades, comprehensive genomic sequence information has become available for an ever-increasing number of species. The development of next-generation sequencing and single molecule, real-time sequencing technologies for RNA sequencing has permitted genome-wide expression analysis in response to various stimuli, providing unparalleled opportunities for biomarker discovery by transcriptomics. However, mRNA levels do not provide a complete picture of cellular function. Most cellular functions such as plant stress tolerance involve multiple interactions of proteins and their metabolites. Furthermore, protein expression levels are dependent not only on transcript levels but also on translational efficiency and regulated degradation (Batelli et al. 2007; Liu et al. 2016b). Finally, proteins function at specific sub-cellular localizations and are susceptible to post-translational modifications (PTMs, often required to enable function) in ways that cannot be predicted from transcript expression levels or from the genomic sequence. Therefore, it is essential to supplement transcriptomics data with direct measurement of protein abundance.

Metabolomics is another important component of “omics” lexicon, providing a global identification and profile of all metabolites (the metabolome) in a given biological system (Dettmer et al. 2007; Alseekh and Fernie 2018; Pinu et al. 2019). It is a rapidly evolving field of research in plant research as changes in metabolite abundance represent the chemical flux generated from various biochemical reactions, molecular mechanisms and biological pathways. Their proximity to phenotype is thought to make them more representative of the cell/organism’s physiological state, more directly reflecting the cascading effects of the environment, gene expression and regulatory processes (Astarita and Langridge 2013; Guijas et al. 2018a). Thus, metabolomics is becoming a powerful tool to study plant molecular phenotypes for plant growth and development, and stress response.

Proteomics and metabolomics both rely on three basic technological cornerstones that include a method of fractionation to simplify complex mixtures; mass spectrometry (MS) to selectively acquire the data needed to identify and quantify individual peptides and metabolites, and bioinformatics analyses to correlate the empirical mass data with genomic or metabolite databases. In the past decade, the advent of high mass accuracy/resolution MS coupled with liquid/gas-chromatography, the development of new bioinformatics tools, and methodological schema for multi-omics integration, have provided not only high-throughput and high quality data generation but also significantly contributed to both biomarker discovery and mechanistic studies in plant research (Feussner and Polle 2015; Ramalingam et al. 2015; Gao et al. 2017; Tang et al. 2020; Pazhamala et al. 2021). Recently, omics research communities have begun to develop MS-based proteomics and metabolomics approaches applicable to the single cell-type and single cell levels. These new developments seek to unravel the unique functions of distinct cell types and/or single cells, despite the tremendous technical challenges involved, including the sensitivity limitations related to the nature of the sample, miniaturization and presence of cell wall in plant single cell analysis (Labib and Kelley 2020; Hu et al. 2021a; Taylor et al. 2021).

**Fig. 1:**
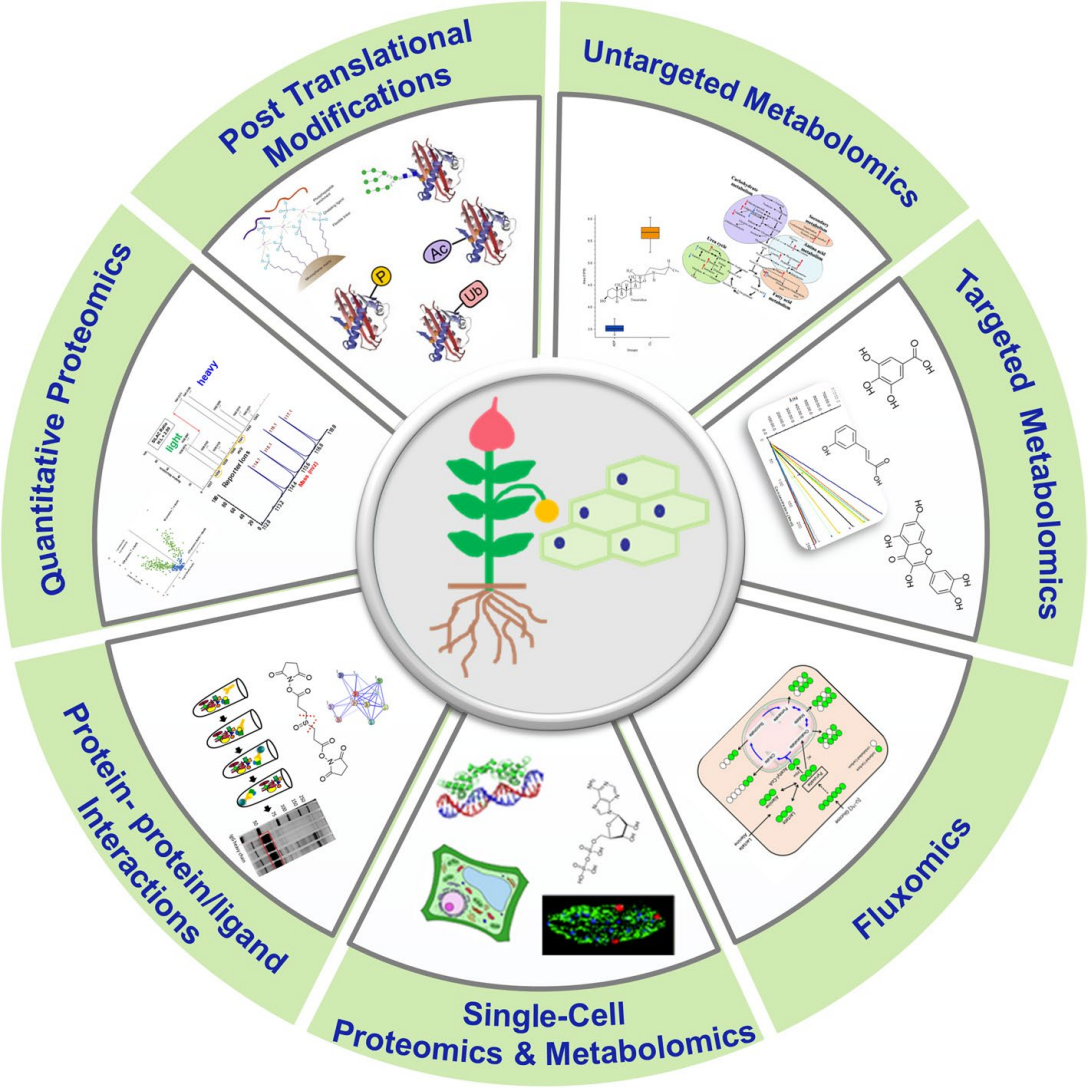
Major types of mass spectrometric based proteomics and metabolomics techniques used for global proteins and metabolites study that are applicable to plant research

In this article, we focus on new advancements in proteomics and metabolomics technologies including discovery proteomics, quantitative PTMs, protein interactions, untargeted metabolomics, fluxomics, targeted metabolomic and their applications in plant biology research. We also highlight the latest developments in single-cell-type and single-cell proteomics and metabolomics in plants as outlined in Fig. 1. We present an intensive review on how these applications are leveraged by the advanced MS technologies and the development of reoptimized workflows that enable omics research in plants.

## Advances in proteomics technologies

### Experimental design, sample preparation, and separation technologies

Over the last two decades, proteomics has been grown in prominence as a strategy to study plant biology (Agrawal et al. 2013; Liu et al. 2019; Jorrin Novo 2021). Similar to other fields (Bhawal et al. 2020; Nakayasu et al. 2021) a rigorous experimental design is essential for meaningful biological interpretation. This requirement includes all phases of the experiment including plant cultivation, application of treatments, choice of plant tissues, etc. (Rose et al. 2004; Atwell 2016). The most critical steps in any proteomics study are protein extraction and sample preparation (Rose et al. 2004; Komatsu and Jorrin-Novo 2021). Given the complexity and diversity of proteins with respect to molecular weight, charge state, hydrophobicity, dynamic range, modification state and cellular distribution, any single extraction protocol will incorporate biases with respect to particular classes of proteins, particularly in whole body or organ extracts. An effective way to minimize this bias is to focus on a subfraction of the proteome by targeting a particular tissue, cell type or subcellular structure (Zhang et al. 2011; Bouchnak et al. 2019). A variety of sample preparation strategies incorporating many separation technologies have been developed for an array of applications. There are two main approaches: an electrophoretic-based approach (also known as a protein-based approach) and a gel-free approach (also called a peptide-based approach). The electrophoretic approach has been largely abandoned as a viable experimental strategy, although it still finds occasional use in a number of niche applications (Jorrin-Novo et al. 2019), it is generally considered an archaic technique putting it beyond the scope of the current work.

Since the first decade of this century chromatographic separation technologies (ultra-high performance liquid chromatography (UHPLC) and associated columns) and MS hardware have made dramatic advances and these have enabled the shift from gel-based to chromatography-based proteomics employing both label-free or label-assisted techniques for quantitation. Most current strategies involve separation/quantification at the peptide level. Complex protein samples are denatured and enzymatically digested, and then the resulting peptide mixture is separated using one or two dimensions of LC (2D-LC). The choice between these two approaches hinges on 1), the complexity of the proteome being investigated and 2), the depth of coverage required. One-dimension approaches have the advantage of speed and utilize a minimum of instrument time but at the cost of reduce proteome coverage and a bias in favor of the largest and most abundant proteins. Two dimensional approaches utilize significantly larger amounts of instrument time and increase turnaround but dramatically increase proteome coverage and minimize the bias for large and abundant proteins. There are several standard approaches to achieve the first dimension of separation that include high pH reversed-phase LC (RPLC), strong cation ion exchange (SCX), hydrophilic-interaction chromatography (HILIC), and affinity chromatography. The second dimension of chromatography is almost always low-pH RPLC coupled directly with tandem mass spectrometry. These approaches have emerged as the preferred tools for protein profiling and PTMs characterization (Ceballos-Laita et al. 2020).

At the whole protein level, affinity purification MS (AP-MS) was a major breakthrough in plant research and is used to investigate protein degradation, PTM identification and localization as well as protein-protein interactions (Bontinck et al. 2018; Zhang et al. 2019). It works on the basic principle of reversible interaction between the affinity ligand and the targeted proteins or specific PTMs. While used less frequently, this approach has been successfully applied at the peptide level as well. A related approach called Immunoprecipitation is a single-step purification that uses an antibody specific for the bait protein or a generic antibody against an exogenous affinity tag covalently linked to the bait protein. This single-step AP can also be performed without the need for specific antibodies such as the streptavidin-binding peptide-tag and His tag, which enable trapping with streptavidin and Ni^2+^ resins, respectively. Hence this technique is also referred as pull-down. Due to its efficiency and simplicity, this approach has gained a growing popularity in plant research, but development of specific antibodies against targeted proteins is expensive and time consuming. As a result, generic antibodies for specific affinity tags including florescent proteins like GFP are most commonly used for purification. One of the major limitations of this approach is the requirement for constructing a recombinant clone expressed in an appropriate host cell. Other limitation includes tedious experimental procedures and non-specific binding that can result in false positive results. Affinity binding is also commonly used as an enrichment method of PTMs like phosphorylation with immobilized metal affinity chromatography (Bontinck et al. 2018). Laser capture microdissection (LCM) is a technique by which cells of a single-type can be harvested from tissue sections visualized under microscope (Chen et al. 2020b). Harvested cells can provide DNA, RNA, and protein for the profiling of genomic characteristics, gene expression, and protein abundance from single-type of cell. Earlier, proteomic analysis of LCM tissues required a larger number of cells, however this problem is dramatically improved with the advent of more powerful separation technologies and highly sensitive mass spectrometers.

### Advanced MS technologies for proteomics

The growing list of applications that are amenable to proteomics has been driven by the rapid advancement of MS technologies over the last decade. The latest Orbitrap Eclipse Tribrid mass spectrometer with advanced quadrupole mass filter, dual-pressure linear ion trap and Orbitrap mass analyzers, is an excellent example (Yu et al. 2020). The system provides maximum analytical capability and flexibility for both top-down proteomics (Kelleher 2004; Cleland et al. 2017) in direct analysis of intact proteins, and bottom-up proteomics for analysis of peptides resulting from the digestion of complex protein mixtures. Bottom-up proteomics has demonstrated broad applications to plant research and is the focus of this review. Another excellent example is the advancement of ion mobility spectrometry (IMS)-based MS such as trapped IMS in timsTOF Pro (Meier et al. 2021), Twave IMS in SYNAPT G2-Si (Hernandez-Mesa et al. 2020) and field asymmetric IMS (FAIMS) in Orbitrap mass spectrometers (Hebert et al. 2018). IMS, as a gas phase “electrophoresis” technique offers rapid structural separation with measured collision cross-section (CCS) values, providing an additional dimension of separation for isobaric molecules and isomers in complex samples (Burnum-Johnson et al. 2019). Thus, IMS-based MS greatly facilitates fast, sensitive and robust proteomics and metabolomics profiling, allowing proteomics for a true high-throughput era. Furthermore, IMS-MS has been demonstrated in contributing greatly to the recent success of single-cell proteomics for increased selectivity by removing singly charged species (Kelly 2020).

**Fig. 2:**
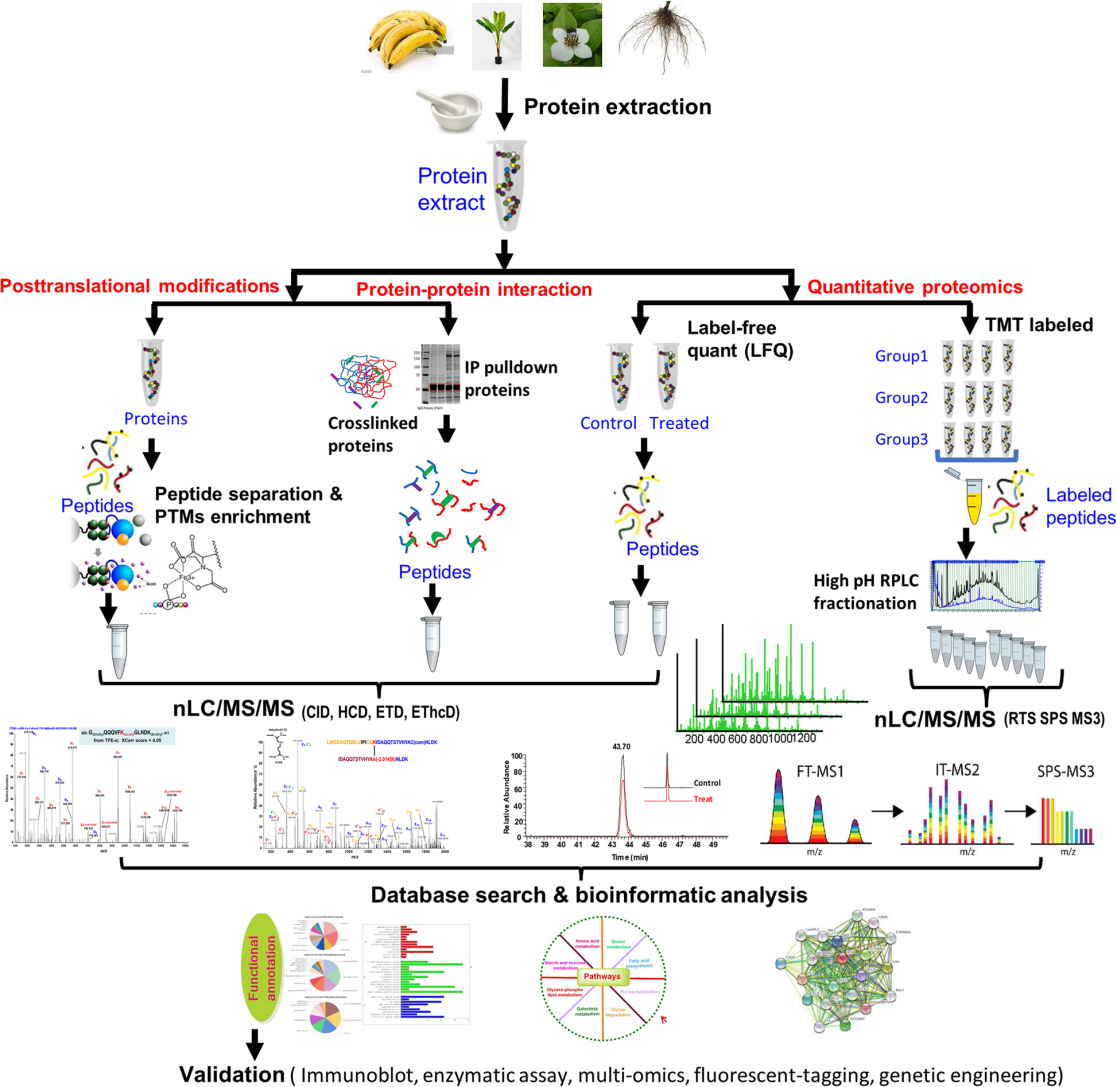
Schematic diagram represents general plant proteomics workflows. Proteins extracted from plant materials are fractionated using either gel-based or gel-free techniques. Three major types of proteomics analysis for identification and quantitation of posttranslational modifications, protein-protein interaction and quantitative proteomics were presented. The MS raw data generated from each workflow using different mass spectrometric techniques are carried out by database search, statistical analysis and bioinformatics analysis. Finally, the outcomes are to be further validated for either generating biological hypothesis or validation of initial mechanistic hypothesis

Effective MS-based proteomics strategies have been developed to address the different biological and analytical challenges depicted in Fig. 2. MS data acquisition for most labeled or label-free proteomics protocols the experiments are carried out in a data dependent acquisition (DDA) mode with dynamic exclusion to minimize the collection of redundant MS spectra (Hart-Smith et al. 2017). DDA defines the maximal scan rate at which mass spectrometers can acquire MS/MS data for near co-eluting peptides. Another data acquisition strategy is data-independent acquisition (DIA) (Zhang et al. 2020a), which has been gaining acceptance in recent years. DIA involves parallel MS/MS analysis of multiple precursor ions simultaneously, allowing for improvements in quantitative reproducibility, depth of proteome coverage while allowing for a *post hoc* targeted interrogation of the data using either *in-silico* fasta database or specially constructed spectral libraries.

#### Quantitative proteomics

One of the advantages of MS-based proteomics is the ability to systematically highlight changes in protein abundance between biological samples through quantitative analysis. Protein abundance difference reflects different regulated states of the cells, a disease state or other biological perturbations such as stress from biotic or abiotic factors or experimental manipulation. Quantitative proteomics for protein abundance profiling is widely-used technique to establish a chemical phenotype associated with a given biological states and to identify biomarkers associated with specific biological state such as disease, abiotic stress. However, it is extremely important to evaluate the replicate datasets to dissect and distinguish biological variations from technical variations in pilot experiments whenever possible. So far either stable isotope-labeling methods or label-free techniques have been frequently applied in quantitative proteomics studies.

The isotopically coded chemical label-based approaches remain most popular in quantitative proteomics due to their multiplexing capabilities which couple high throughput, quantitative accuracy and compatibility with 2D liquid chromatography tandem mass spectrometry (LC-MS/MS) analysis. Chemical labeling using isotope coded isobaric reagents like tandem mass tag (TMT) (Thompson et al. 2003) and Isobaric tags for relative and absolute quantification (iTRAQ) (Ross et al. 2004) represent a high-throughput and robust technique that requires further discussion. They have emerged as one of the most widely-used techniques in quantitative shotgun analysis that is particularly useful for global proteome profiling and associated functional changes in plants. The isobaric tag consists of an amine-specific reactive group for labeling free amine groups of peptides, a balance group and a reporter group released under MS2 or MS3 fragmentation that provides mass signature for relative quantitation. The major advantage of TMT labeling is that it can afford a high degree of multiplexing (up to 18 plex) since it is able to monitor up to 18 samples in a single analysis (Li et al. 2021b). The main disadvantage of MS2-based quantitation is the co-isolation and co-fragmentation of near isobaric labeled ions, which causes a ratio distortion problem (Ting et al. 2011). This distorted ratio issue can be dramatically reduced using synchronous precursor selection (SPS) MS3 method (McAlister et al. 2014) in which multiple MS2 fragment ions (acquired for peptide identification) are further fragmented in MS3 yielding a reporter ion population without the interfering signals. However, due to the extended cycle time associated with the inclusion of an MS3 scan, the SPS MS3 approach results in a decreased spectral acquisition rate and a reduction in the number of proteins quantified of ≈ 30% compared to traditional MS2-DDA analysis. To maintain in-depth proteome coverage with accurate and precise quantitative profiling, a real-time search (RTS) SPS MS3 method has been developed recently (Erickson et al. 2019). RTS SPS MS3 approach carries out MS2 spectral identifications in real time within milliseconds so that only identified peptides can trigger quantitative MS3 scan, which increases the number of productive precursors that are subject to MS3 offsetting the longer cycle time and resulting in enhanced proteome coverage and accurate TMT quantitation (Fu et al. 2021).

Another popular labeling approach is stable-isotope labelling by amino acids in cell culture (SILAC). This has been covered in literally many excellent prior reviews of this subject (Gruhler et al. 2005; Matthes et al., 2014). Few studies have been done with SILAC labeling in plant research, due to the requirement for metabolically labeling under culture conditions. So far, most SILAC-based labeling proteomics studies have been done to study the formation of signal-dependent protein complexes, and modification-dependent protein-protein interactions in *Arabidopsis thaliana* seedlings by SILAC, but suboptimal labeling efficiency have been known to compromise quantitation (Gruhler et al. 2005; Thelen and Peck 2007; Schutz et al. 2011).

Despite the popularity of label-based methods, the main limitations of the label methods include cost of isotopic labeling and increased complexity of the experimental procedures which can lead to sample loss and add experimental variations. As a result, label-free quantitation (LFQ) approaches have recently been widely used as alternatives. In LFQ analysis, the intensities of identical peptides from two or more samples can be compared directly by traditional DDA method. However, recent developments in bioinformatics software allows DIA to be comparable with DDA in the number of peptide identifications for label-free samples while still allowing precise quantification (Hu et al. 2016). The improved protein coverage by DIA makes it possible to carry out label-free quantitative analysis of very complex samples (Rosenberger et al., 2014).

#### Protein PTMs: plant phosphoproteomics

PTMs are important chemical changes of proteins that increase proteome diversity tremendously and allow for effective regulation of cellular processes. More than 400 distinct types of PTMs have been found which have been shown to impact protein function (Khoury et al. 2011). Therefore, the identification and mapping of PTMs are important activities because they help to define the proteome in terms of “proteoforms”. These PTM studies typically require an enrichment step due to the low stoichiometry of most PTMs. The presence of PTMs is implied by specific mass shifts, thus the specific site of the modification can be deduced through the analysis of fragmentation data (MS^2^-MS^n^). Historically, CID or HCD fragmentation has been used to analyze PTM peptides. However, several non-ergodic fragmentation strategies (ETD, ECD, etc.) have proven valuable for the analysis of labile PTMs (Chi et al. 2007).

The most extensively studied PTM types are phosphorylation (on S/T and Y residues), acylation (acetylation, succinylation and malonylation on K), ubiquitination (K) and glycosylation (N, S/T and hydroxyproline) (Ramazi and Zahiri 2021). Phosphorylation is one of the most important and well-studied reversible PTMs in plants. Kinases catalyze protein phosphorylation by transferring a phosphoryl group typically from ATP or ADP to the hydroxyl group of S/T/Y residues, but phosphorylation on several unusual residues such as His, Asp, Cys, Arg and Lys has been reported (Hardman et al. 2019). Phosphatases are responsible for removing the phosphor group from the modified residues. Phosphorylation in plants regulates a wide range of cellular processes such as transmembrane signaling, intracellular amplification of signals, and cell-cycle control including hormone sensing and environmental stress responses (Schulze 2010; Ingelsson and Vener 2012). It often leads to protein structural changes that can directly modulate protein activity, and induce changes in interaction partners or subcellular localization (Li et al. 2015b). Plant genomes encode twice the number and diversity of kinases compared with mammalian genome, indicating the importance of the plant phosphoproteome in regulating responses to both abiotic and biotic stresses (Zulawski et al. 2013; Silva-Sanchez et al. 2015).

Considerable analytical challenges remain for the study of PTM in plants particularly in phosphoproteomics due to a number of factors including the high dynamic range and complexity of the plant proteomes, the unique challenges associated with the plant cell walls, and the interference from chlorophyll and secondary metabolites. A universal protocol incorporating optimized protein extraction procedures involving guanidine hydrochloride, methanol-chloroform precipitation, and phase-transfer surfactant assisted tryptic digestion has been reported recently. This new approach was found to increase the coverage of tomato phosphoproteome more than 2-fold compared with the standard protocol (Hsu et al. 2018). In the past decade, many phospho-enrichment strategies have been developed to enhance the identification of low abundant phosphopeptides and phosphoproteins (Batalha et al. 2012; Silva-Sanchez et al. 2015). Immobilized metal affinity chromatography (IMAC) is often coupled with strong cation exchange (SCX) for two-step phosphopeptide enrichment in large-scale phosphoproteomic studies. However, more recently the use of metal dioxide affinity chromatography such as titanium dioxide (TiO_2_) and zirconium dioxide (ZrO_2_) has become more common. A serial enrichment procedure with both TiO_2_ and ZrO_2_ has been shown to increase the efficiency of capturing phosphopeptides (Gates et al. 2010). A combination of TiO_2_ enrichment and HILIC fractionation for subsequent LC-MS/MS analysis resulted in the identification of 1500 phosphopeptides from 685 phosphoproteins in the leaves of two varieties of wheat that suggested differential regulation of the phosphorylation status of signaling proteins, transcription and translation regulators, and membrane-associated proteins (Yang et al. 2013a). Given the required enrichment steps, most phosphoproteomics studies are conducted using label-free LC-MS/MS workflows (Engelsberger and Schulze 2012; Wang et al. 2013a; Qiu et al. 2016; Ford et al. 2020; Li et al. 2021c). However, iTRAQ/TMT labeling has also become popular in plant phosphoproteomics (Yang et al. 2013a; Fan et al. 2014) due to their shared capability to multiplex up to 18 samples in a single experiment for both enrichment and LC-MS/MS analysis. Furthermore, this approach allows for parallel global proteome analysis using either a TiO_2_ flow-through fraction (Yang et al. 2013a) or a small aliquot of the sample for the quantitative work while reserving the majority of the sample for enrichment and phosphopeptide analysis (Yang et al. 2018). Parallel proteomics and phosphoproteomics analyses allow one to distinguish the changes for each of the phosphorylation sites of a given protein from the change of that proteins general abundance and is particularly useful for determining the biologically important sites of proteins that contain multiple phosphorylated sites with different degrees of change.

It should be pointed out that identifications, localizations, and quantifications of different combinations of PTMs on the same protein (various proteoforms) are generally difficult by traditional bottom-up proteomics. However, both top-down proteomics for direct analysis of whole proteins and middle-down proteomics for analysis of large peptides by limited proteolysis can be used to detect multiple co-occurring PTMs in a specific proteoform and a large peptide, respectively (Leutert et al. 2021). Top-down proteomics for characterization of proteoforms has been well covered by several recent review articles (Schaffer et al. 2019; Carbonara et al. 2021; Melby et al. 2021). We will omit this topic and direct the interested reader to these.

#### Protein-protein interactions and protein complexes

Protein–protein interactions (PPIs) are fundamental to all biological processes (Cusick et al. 2005). Vital cellular functions such as DNA replication, transcription and mRNA translation, require the coordinated action of many proteins that are assembled into an array of multi-protein complexes of distinct composition and structure. Many important biological processes in plants such as organ formation, homeostasis control, plant defense, signal transduction and stress response are comprised of, and regulated by, dynamic signaling networks of interacting proteins that directly or indirectly respond to specific effector molecules (Bontinck et al. 2018; Struk et al. 2019). Since almost all proteins interact with other molecules a comprehensive determination of PPIs within an organism is an essential aspect of systems biology that is used to uncover unknown functions and to gain insight into complex cellular networks. However, understanding the dynamic nature of protein complexes with respect to composition and stability and cellular state presents a significant challenge. Many different methods for determining PPIs have been developed and the topic has been well-reviewed (Struk et al. 2019). These include the yeast two hybrid (YTH) system, the first technique used for large-scale interactome maps (Uetz et al. 2000), affinity purification coupled to MS (AP-MS) (Gingras et al. 2007), proximity labeling coupled to MS (PL-MS) (Kerbler et al. 2021) and bimolecular fluorescence complementation (Miller et al. 2015). Substantial advances in determining composition, regulation and function of molecular complexes have been obtained by MS-based proteomics (called interaction proteomics or interactomics) leading to a greater understanding of the molecular basis of complex biological processes (Aebersold and Mann 2016).

Affinity purification and mass spectrometry (AP-MS) is one of the enabling developments for PPI studies. Targeted proteins complexes are isolated from plants using antibodies against either the protein of interest or a tagged protein, which is often called co-immunoprecipitation (Co-IP). It has the great advantage of capturing the physiological state, abundance, and interactions of the targeted protein without the need for cloning or overexpression. The protein complexes isolated by Co-IP are then eluted and analyzed by LC-MS/MS (Fukao 2012). These affinity-based methods have improved greatly because of the development of highly sensitive MS instrumentation and novel bioinformatics approaches (Armean et al. 2013; Qu et al. 2017). To minimize the impact of non-specific binding, a second purification step has been introduced by means of a double affinity tag. One of the most frequently applied tandem affinity purification tags in plant research is the GS tag and its derivatives (Van Leene et al. 2007). The GS tag consists of two immunoglobulin domains of protein G and a streptavidin-binding peptide separated by a cleavage site. AP-MS is used to study plant growth and development in the relevant biological contexts, such as specific plant organs, for example, flowers, leaves and roots and provides an enhanced view of the protein complex composition (Batelli et al. 2007; Chang et al. 2009). A combined AP-MS with an LFQ method has been developed (Keilhauer et al. 2015) and well described in a recent review article (Kerbler et al. 2021). This has become a common approach to differentiate true interactors from the background. In this method, the increased amounts of unspecific binding proteins can be advantageous, because they are used in the postprocessing pipeline for a more exact normalization and as a kind of quality control. The LFQ combined with AP-MS can also be used to assess the dynamics of PPIs during cellular signaling or after cellular perturbations, because protein complexes copurified with the same bait under two different conditions can be compared in a quantitative manner. The Co-IP allows for the identification of PPIs in certain tissues or during specific developmental stages and in different genetic backgrounds and is also considered one of the standard methods for PPI validation. However, this AP-MS technique does not provide information about the direct interaction between proteins but rather about their coexistence in a higher order protein complex (Xing et al. 2016). The topology of the protein interactome is not achievable from the AP-MS strategy. PL-MS uses enzymes that produce reactive molecules for covalently interacting with proteins in close proximity. Although the use of PL-MS in plant still remains its infancy, recent development of new proximity labeling enzymes TurboID in planta (Zhang et al. 2020b), pupylation-based interaction tagging (Pup-IT) for PPIs at membranes (Siva Sankar and Dengjel 2021) and limited proteolysis-MS (LiP-MS) (Pepelnjak et al. 2020) for protein-small molecule interactions has considerably expanded its applications in plants.

Another development for the identification of PPIs in plants involves cross-linking mass spectrometry (XL-MS) (Zhu et al. 2016a; Liu et al. 2018). Chemical cross-linking followed by mass spectrometry analysis enables identification of proximal amino acid residues within protein complexes, providing vital insights into the structure and interactions of proteins/protein complexes (Chavez and Bruce 2019). Notably, the recent development of a MS-cleavable cross linkers such as disuccinimidyl sulfoxide (Kao et al. 2011) and disuccinimidyl dibutyric urea (DSBU) (Ihling et al. 2020) allows to cleave cross-linked peptides during MS/MS for subsequent MS3 acquisition of cleaved peptides, which facilitates peptide identification using traditional database based approaches (Liu et al., 2015) and allows multiplexed quantitative XL-MS (Yu et al. 2016). In addition, hydrogen–deuterium exchange mass spectrometry (HDX–MS) is able to determine the interaction surfaces and solvent-exposed regions and is emerging as a powerful methodology to study protein dynamics, protein folding, protein-protein interactions, and protein-small molecule interactions (Masson et al. 2019; Li et al. 2020d; Gutkowska et al. 2021).

The latest development of thermal proteome profiling (TPP) technology can also be used for interrogating protein-protein interactions (Mateus et al. 2020). This TPP was initially developed for drug discovery in screening of the targeted proteins by the known ligands (drugs) under a more physiologically relevant environment such as intact live cells level (Savitski et al. 2014). The basic concept of TPP is that proteins become more resistant to heat-induced unfolding when complexed with a ligand or other macromolecules. Combining the principle of the cellular thermal shift assay (TSA) with multiplexed quantitative MS such as TMT10-plex compared to the lysate TSA, TPP allows for detecting protein thermal stability (melting temperature, *Tm*) on a proteome-wide scale (Franken et al. 2015). TPP is becoming a powerful tool for detecting a wide range of physiological changes in protein state: protein-metabolite interactions, post-translational modifications, protein-protein and protein-DNA interactions. (Mateus et al. 2020) and an example in plant has been reported in *Arabidopsis thaliana* (Volkening et al. 2019). However, its limitations include: 1) requirement of substantially changed percentage of the population of any single protein sequence to be reflected in its altered *T*m, which will lead the induced change of *T*m by most PTMs with low stoichiometry difficult to be detected; 2) no information on domain change and what amino acids responsible for identified proteins with altered *Tm*. Therefore, TPP is best used in conjunction with other structural proteomics methods (Blackburn et al. 2022).

### MS data processing, assembly and bioinformatics

A key advancement in MS-based proteomics was the development of algorithms in database search software for peptide identifications (which infer protein identity) by matching the observed masses of precursor and fragment ions with those predicted from a sequence database. The algorithms allow for automated interrogation of genomic databases with acquired large MS and MS/MS datasets using predetermined parameters and other search criteria to generate lists of putative peptide spectrum matches. As genome-wide next-generation sequencing and RNA sequencing technologies continued to advance, the number of species with fully sequenced genomes has exponentially increased, including 341 plant genomes (https://www.ncbi.nlm.nih.gov/genome) and 181 horticultural species (Chen et al. 2019). Thus, the lack of databases is no longer a major bottleneck in most plant proteomics research. Most protein sequence databases derived from plant genomic sequences can be downloaded from NCBI Viridiplantae, GenBank, DDBJ, and UniProt. Several specific sequence retrievals can also be performed from databases dedicated to plants such as Phytozome, plaBi, and Gramene database, which are subsets of the Ensembl Plants database and PlantGDB. However, large proteomics datasets acquired by rapidly evolving MS technology with different acquisition workflows employing multiple fragmentation methods present a number of challenges to determine the correct peptide assignments to MS/MS spectra. These challenges require that extremely powerful search algorithms be constantly enhanced and developed to take full advantage of the data acquisition technology. **Table** **1** shows a partial list of commonly used database search software having the search engines tools for DDA proteomics raw files from five major mass spectrometer vendors (Thermo, ABSciex, Waters, Bruker and Agilent) and 10 public available search tools such as MaxQuant and Mascot etc.. The search engines and software tools specifically for DIA proteomics datasets were extensively described in a recent review paper (Zhang et al. 2020a). To properly interpret the protein identification in particularly large shotgun proteomics dataset against a large database, it is necessary to have a reliable estimate of the false discovery rate (FDR), which is a measure of the percentage of putative protein identifications that are likely to be false. Almost all database search algorithms have integrated a target/decoy strategy for determining the FDR with Benjamini–Hochberg procedure. Even with the high quality of MS and MS/MS spectra acquired by high mass accuracy/resolution instruments, it is necessary to set up an FDR threshold in effectively controlling the number of false positives in proteomic data (Choi and Nesvizhskii 2008).

**Table 1:** A partial list of commonly used database search software tools for proteomics analysis

Software name	Latest version	Source availability	Developer	URL and references
Proteome Discoverer	2.5	proprietary	Thermo Scientific	https://www.thermofisher.com/order/catalog/product/OPTON-30810
ProteinPilot	5.0.2	proprietary	Sciex	https://sciex.com/products/software/proteinpilot-software
ProteinLynx Global Server	3.0.3	proprietary	Waters	https://www.waters.com/waters/en_US/ProteinLynx-Global-SERVER-(PLGS)/nav.htm?cid=513821&locale=en_US
PaSER	2022	proprietary	Bruker	https://www.bruker.com/en/products-and-solutions/mass-spectrometry/ms-software/paser
MassHunter	11.0	proprietary	Agilent	https://www.agilent.com/en/product/software-informatics/mass-spectrometry-software/data-analysis
MASCOT	2.8	proprietary	Matrix Science Inc	https://www.matrixscience.com/server.html; (Perkins et al. 1999)
MaxQuant	2.0.3.0	Freeware	Max Planck Institute of Biochemistry	https://www.maxquant.org/; (Cox and Mann 2008)
Byonic	4.2	proprietary	Protein Metrics Inc	https://proteinmetrics.com/byos/; (Bern et al. 2007)
Scaffold	5.1.0	proprietary	Proteome Software, Inc.	https://www.proteomesoftware.com/products/scaffold-5;(Searle 2010)
MSFragger	3.4	Freeware	University of Michigan	https://www.nesvilab.org/software.html;(Kong et al. 2017)
OMSSA	2.1.19	Freeware	The NIH intramural research program	https://ftp.ncbi.nlm.nih.gov/pub/lewisg/omssa/;(Geer et al. 2004)
Phenyx	Phenyx ®	proprietary	Geneva Bioinformatics (GeneBio)	https://ionsource.com/functional_reviews/Phenyx/phenyx-web.htm; (Colinge et al. 2003)
PRAKS DB	Xpro	proprietary	Bioinformatics Solutions Inc.	https://www.bioinfor.com/;(Zhang et al. 2012)
Protein Prospector	6.3.1	open source	University of California, San Francisco	https://prospector.ucsf.edu/prospector/mshome.htm;(Chalkley et al. 2005)
X!Tandem	2017.02.01	open source	University, Ghent, Belgium	http://www.thegpm.org/TANDEM/index.html;(Muth et al. 2010)

The diversity in data-analysis strategies from different types of mass spectrometers including various outputs leads to big challenges for the computational analysis of MS data that often leads to substantial differences between results obtained with different software tools. Therefore, some “3rd party” software tools were developed enabling analysis of raw data files from multiple vendors’ platforms to address some of these challenges. Among them, MASCOT (Perkins et al. 1999) and MaxQuant/Andromeda (Cox and Mann 2008) are the most widely used database searching tools for large-scale proteomics data. Mascot used an additional Distiller algorithm for label-free proteomics. The universal free software, MaxQuant using its own Andromeda search engine for peptide identification (Cox et al. 2011; Valikangas et al. 2018) is applicable for mostly label-free and labeled quantifications from high resolution data files, OpenMS, an open-source software platform is another tool providing a highly flexible and professional software environment equally suited for end users (Rost et al. 2016). Peptide identifications were performed within PEAKS software, another vendor-neutral tool also using its own search engine PEAKS DB combined with PEAKS *de novo* sequencing (Zhang et al. 2012). An additional Peaks Q module allows for relative protein abundance changes across a set of samples simultaneously. For quantitative proteomics, due to different strategies or workflows used for data acquisition, more suitable tools were developed, such as PyQuant and SILVER for stable isotope labeling quantification, RIPPER and LFQuant for label-free quantification (Chang et al. 2014; Mitchell et al. 2016; Van Riper et al. 2016). Most recently, another efficient quantitative software PANDA was developed that supports both label free and labeled quantitation with existing peptide identification tools and accurate quantitation (Chang et al. 2019).

Following data processing, database search and statistical analysis for discovery of candidate proteins and/or their modifications or interacting complexes, further bioinformatics analyses are required for functional annotation of those protein candidates. The most widely used functional annotation is ‘Gene Ontology’ (GO) having three separate GO terms as biological process, cellular component, and molecular function respectively, along with pathway enrichment analysis. A biological pathway is a series of reactions within the cell that exert a specific biological function. The proteins that are directly involved in reactions plus those that regulates the pathways belong to pathway databases. Some resources and databases available for the protein pathways such as KEGG, Ingenuity and Pathway Knowledge Base Reactome are the most often used pathway databases with a comprehensive data for protein metabolism, signaling and interactions. Perhaps the best-known software for automated functional annotation pipeline is BLAST2GO (Conesa and Gotz 2008) that also incorporates InterProScan for protein family classification and KEGG data. In functional analysis involved in cellular signaling, plant phosphorylation site databases including PhosPhAt (Durek et al. 2010) for *Arabidopsis*, Plant Protein Phosphorylation DataBase (P3DB) (Gao et al. 2009) for 45 plant species (https://www.p3db.org/home) and PHOSIDA (Gnad et al. 2011) can be used for predicting phosphorylation sites with an average predicting accuracy of 82.4% for pSer, 78.6% for pThr, and 89.0% for predicting pTyr by PlantPhos tool (Lee et al. 2011). For protein complex studies, STRING is not only a widely used database with wealthy protein interaction data, but also it connects to various other resources for literature mining. Protein networks can be acquired based on the list of proteins/genes provided and the available interactions using the STRING database (https://string-db.org/). In addition, Biological General Repository for Interaction Datasets (BioGRID) contains a large collection of protein–protein interactions for all major model organism species and humans (Chatr-Aryamontri et al. 2017). Another popular tool is Skyline, an open source software developed for targeted proteomics data analysis (Pino et al. 2020) over the past decade, but recently it becomes available for targeted metabolomics data analysis (Adams et al. 2020). Notably, Skyline enables to support almost all of data analysis workflows such as SRM/MRM, PRM, DIA and targeted DDA.

## Proteomic applications in plant research

### Mechanistic understanding of plant stress tolerance

Plants are constantly affected by abiotic and biotic stresses during growth, development and adaptation to their environment. Plant proteins and metabolites play an important role in the maintenance of cellular homeostasis, and regulate physiological changes to better adapt to prevailing environmental stresses. The plant immune system responds to biotic stress as a complex system with interactions and crosstalk between multiple signaling pathways characterized by various signaling proteins and with a diverse set of stress-related proteins. Therefore, protein profiling under various stress conditions has been extensively investigated (Kosova et al. 2018; Liu et al. 2019). Quantitative proteomics provides comprehensive analysis of proteins allowing for the identification of key metabolic pathways affected by biotic or abiotic stress. iTRAQ labeling in proteomics enables to analyze and quantify up to eight phenotypes with high resolution (Pierce et al. 2008), and is widely used in model plants such as *Arabidopsis* (Lan et al. 2011) and rice (Wang et al. 2014b) but also has provided a platform to profile and understand the non-model species through comparative proteomics (Yang et al. 2011; Zhou et al. 2016a). We are one of the earlier groups applying iTRAQ-based quantitative proteomics to investigate the temporal responses of plantain (*Musa spp.* Dajiao; ABB Group) proteome to identify the proteins related to the cold stress as Dajiao has superior cold tolerance compared with Cavendish Banana (*Musa spp.* Cavendish; AAA Group), an important tropical fruit with high economic value (Yang et al. 2012). The global proteome results suggest that an increase in antioxidant capacity via adapted ROS scavenging capability, reduced production of ROS and lipid peroxidation contributes to molecular mechanisms for the increased cold tolerance in plantain. Proteomic profiling and identification of some membrane proteins has great potential value for developing cold tolerant banana cultivars. Further iTRAQ analysis of the membrane proteomes of both Daojiao and Cavendish Banana under cold stress showed membrane-bound proteins such as peroxidases and aquaporins that were consistently induced at an early stage of cold stress (He et al. 2018). After cross-verification by qRT-PCR and MRM-targeted quantitation, and fluorescent-based subcellular localization analysis, the authors concluded that 2 peroxidases, and 5 aquaporins are mainly involved in decreased lipid peroxidation and maintaining leaf cell water potential, which appear the key cellular adaptations contributing to the cold tolerance of Dajiao (He et al. 2018). These proteomics findings provided a good complement to the transcriptomics datasets for Dajiao’s high cold tolerance and its mechanisms (Yang et al. 2015). Meanwhile, a similar iTRAQ approach was used for discovery of the key ergosterol biosynthesis pathway to the conidial germination of the soilborne fungus *Fusarium oxysporum f. sp. cubense* tropical race 4 (*Foc* TR4), a most important lethal disease of Cavendish banana (Deng et al. 2015). This finding led to the successful development of transgenic bananas with superior resistance by host-induced gene silencing of two ergosterol biosynthesis genes (*ERG6/ERG11*) in *Foc* TR4 (Dou et al. 2020), which lays the groundwork for disease-resistance breeding in bananas and possible other crops.

Since poor correlation is often found between gene expression levels and protein abundances in organisms under abiotic and biotic stresses, proteomics has thus become the preferable strategy to identify underlying key factors and metabolic pathways (Feussner and Polle 2015), which is proven as an effective approach to identify candidate proteins in response to cold stress in plant species (Zhang et al. 2016; Gao et al. 2019). Jiang et al. conducted a TMT10plex-based global proteome analysis for the leaves of *Citrus junos* seedling under the cold stress. These authors identified over 400 proteins accumulated in seeding leaves that are mainly related to the starch and sucrose metabolism as well as secondary metabolism. After physiological analysis, the authors propose that enhanced sugar and secondary metabolisms are the potential factors underlying the response of citrus rootstock to cold stress (Jiang et al. 2021). Qin et.al. performed root proteome analysis by the TMT-based quantitative methods for the characterization of proteins in rapeseed to identify the mechanisms underlying rapeseed root adaptions to nitrogen deficiency (Qin et al. 2019). Proteins involved in cell wall organization or biogenesis were observed in high abundance, while most identified peroxidases were reduced in the N-deficient roots. Peroxidase activities were found decreased, which might promote root elongation while lowering the solidity of N-deficient roots. In recent plant proteomics analysis, LFQ approach particularly based on DIA method has been increasingly used for global quantitative profiling that provides insights into many processes like stress response and tolerance, nutrient sensing and development. Several proteomic studies have reported in-depth identification of differentially expressed transcription factors responsible for the development of fruits or other organs of tissues at various stages of development. One such study was performed using DIA for two winter rapeseed cultivars, one with cold tolerance and another with cold sensitive, and these authors discovered that the cold tolerance is related to reactive oxygen species (ROS) scavenging, possibly through metabolic pathways including flavonoid and ubiquinone biosynthesis, and other terpenoid-quinone biosynthesis (Mi et al. 2021). Li et al. conducted a global proteomics analysis for *Morus alba* leaves under high level ultraviolet-B (UV-B) radiation and dark incubation (UVD) by SWATH-based DIA quantitative analysis. Other than significantly increased photorespiration in UVB group and phenolic compounds in UVD group, the abundances of proteins involved in the ubiquitin-proteasome system (UPS) and antioxidative enzyme activities were significantly increased in both UVB and UVD groups, suggesting UPS related proteins participated in the resistance to UV-B radiation through abscisic acid (ABA) signaling and protein degradation (Li et al. 2022b). Another DIA-based LFQ investigation of the global proteomes of WT tomato fruit and its *cd2* mutant was conducted to identify tomato proteins regulated by the *CUTIN DEFICIENT2* transcription factor and involved in cutin biosynthesis (Martin et al. 2016).

Quantitative proteomics for global PTM analysis is a fast-growing field that provides new insights into the regulatory roles of protein PTMs in cellular metabolic networks and has been widely used for probing stress tolerance in plants (Liu et al. 2019). Protein phosphorylation is an important signaling mechanism underlying the plant response to biotic and abiotic stress (Rampitsch 2017; Liu et al. 2019). Most studies in plants have been focused on protein kinases and identification of the phosphorylated substrates. For example, many plant kinases are activated and positively regulate plant frost tolerance at the post-translational level. The mitogen-activated protein kinases (MAPK) constitute one of the most important signaling mechanisms in plants, and plays essential roles in enhanced frost tolerance (Furuya et al. 2013; Gao et al. 2017). In a TMT-based comparative phosphoproteomics analysis of Daojiao and Cavendish bananas under cold stress, the phosphorylation level of Thr31 on MAPK kinase 2 (MKK2) was increased significantly in the cold-tolerant Dajiao cultivar along with decreased MKK2 abundance for a time course of cold stress. Meanwhile, no detectable T31 phosphorylation with increased abundance of MKK2 protein was found in the cold-sensitive cultivar, Cavendish (Gao et al. 2017). These findings provide new evidence that the signaling pathway of cellular MKK2 phosphorylation is associated with the molecular mechanisms of high tolerance to cold stress in Dajiao. Tan et al. reported a parallel proteome and phospoproteome profiling of *Arabidopsis* seedlings under short-time cold stress using a DIA-LFQ analysis. These authors found a rapid (within 2 h of cold stress) and broad change of phosphorylated peptides from >1200 proteins that includes >140 kinases, >40 transcriptional factors and >40 E3 ligases. Those early response proteins to cold stress were linked to phospholipid signaling, cytoskeleton reorganization, calcium signaling, and MAPK cascades (Tan et al. 2021). In the plant target of rapamycin (TOR) kinase, a conserved serine/threonine protein kinase was found to play an essential role in maintaining cellular homeostasis. A combined quantitative phosphoproteomics analysis involving a targeted TOR complex in *Arabidopsis thaliana* has been recently reported to not only detect TOR-regulated phosphoproteins linked to the TOR signaling network but also enabled the identification of candidate TOR substrates (Van Leene et al. 2019). Phosphorylation is not only responsible for many biological processes in plants, but also often functions in coordination with other PTMs, resulting in crosstalk between PTMs on the same protein. O-GlcNAcylation and phosphorylation are examples that occur at the same amino acid sites/residues and are involved in the regulation of several cellular processes such as transcription, cell signaling, hormone sensing and others (van der Laarse et al. 2018).

### Signaling pathways exploration by protein interactomics

Mapping protein-protein interaction (PPI) networks and their dynamics is fundamental in understanding protein function and signaling transduction in cellular activities. *Arabidopsis thaliana* is the well-studied model plant for PPI with 95,382 PPIs being published for 12,617 proteins (approximately 46% of *Arabidopsis* genes coding for proteins) and deposited in databases being used as the basis for a Cytoscape network (Yilmaz et al. 2022). AP-MS is one of the popular approaches to study many aspects of plant cellular processes including plant growth and development. Nee et al. used an AP-MS approach for uncovering the role of GERMINATION 1 (DOG1) and its regulatory mechanisms underlying *Arabidopsis* seed germination. The *GFP* tagged *DELAY OF GERMINATION 1 (DOG1)* transgenic lines were constructed and used for IP pulldown of DOG1 interacting complexes of native seed protein extracts that were subjected to subsequent protein identifications by LFQ-MS analysis (Nee et al. 2017). Four phosphatases: AHG1, AHG3, RDO5 and PDF1 were found among the proteins that intact with DOG1 in seeds while two of them: AHG1 and AHG3 are Clade A type 2C protein phosphatases (PP2Cs) and essential for DOG1-dependent control of seed dormancy. In combination with genetic analysis, the authors found that the interaction of DOG1 with AHG1 and AHG3 can negatively affect the function of these PP2Cs rather than that these phosphatases control DOG1 activity by phosphorylation (Nee et al. 2017).

In planta chemical cross-linking MS (XL-MS) has emerged as an alternative approach for mapping PPIs and studying protein complexes (Zhu et al. 2016a; Liu et al. 2018). Liu et al. developed a chemical cross-linker, azide-tag-modified disuccinimidyl pimelate that was used in planta for chemical cross-linking within *Arabidopsis* tissue, followed by streptavidin enrichment of the biotin-tagged cross-linked peptides, LC-MS/MS analysis, and the use of specialized software (ECL2 and SQUA-D) to identify and quantify cross-linked peptides (Liu et al. 2018). A total of 354 unique cross-linked peptides were identified with 61 representing the inter-protein crosslinks including a conserved protein family: prohibitins (PHBs) that are related to cell proliferation, saline and oxidative stress (Wang et al. 2021a). The PHB3–PHB6 protein interaction was confirmed by Co-IP and super-resolution microscopy experiments (Liu et al. 2018). Recently, a MS-cleavable cross linkers DSSO has been applied for intermolecular and intramolecular interactions of the *Arabidopsis* plasma membrane proton pump (H^+^-ATPase), an essential enzyme for cell surface energetics, regulation of cell elongation and response to abiotic and biotic stimuli (Nguyen et al. 2020). The strep-HA-tagged *Arabidopsis* H^+^-ATPase 2 (AHA2) expressed in yeast under both N14 and N15 media was purified on streptactin resin prior to DSSO crosslinking reaction, and the C-terminal domain of AHA2 was found to be extensively crosslinked to other domains in intramolecular monomer as well as intermolecular interactions through observed mixed-isotope cross-linking pairs. The results not only suggest the regulatory role of C-terminal domain dynamic interaction in the catalytic activity of AHA2, but also support an Interface structure between monomers of AHA2 based on many intermolecular crosslinks found in the cytoplasmic domain (Nguyen et al. 2020). Another recent development for plant PPI application is the combination of crosslinking and tandem affinity purification coupled to MS (XL–TAP–MS) to address the big analytical challenge for detection of low-abundance protein complexes and in vivo protein–protein interactions in complex biological samples. Leissing et al. used an in vivo-biotinylated protein domain flanked by two hexahistidine sequences for affinity isolation of formaldehyde–crosslinked protein complexes of the MKK2-MPK4 signaling module in *Arabidopsis* (Leissing et al. 2021). Out of 107 proteins identified as putative interactors of the MKK2–MPK4 module, 9 are interacted specifically with MKK2, 47 with MPK4, and 51 interactors are co-purified with both bait proteins. More importantly, many of the module-interacting proteins are involved in abiotic stress signaling and various biotic responsive pathways in *Arabidopsis* (Leissing et al. 2021).

### Functional characterization of cell-specific proteins by single-cell-type proteomics

Studies conducted using standard bottom-up protocols generally involve the use of bulked tissue or organ samples containing uncharacterized mixtures of diverse and intermingled cell types, each with unique proteomes optimized for specific sets of biological functions. Studies of these bulked samples capture only the weighted population mean of protein expression and obscure important information concerning intercellular heterogeneity as well as all spatial effects. It is becoming increasingly clear that a very granular sampling strategy is required to provide the high spatial resolution and cell specific proteomic information required to facilitate the disambiguation of the biological complexity that underlie a plants response to biological stimuli.

Considerations of this kind have given rise to a growing effort to develop methods to carry out comprehensive, quantitative proteomics studies on single or small numbers of cells and make these accessible to all researchers. While other non-MS centric methods have been utilized (Seyfferth et al. 2021; Shaw et al. 2021; Cuperus 2022), single cell proteomics by MS seems positioned to revolutionize our understanding of cellular functions and regulatory networks. In the past decade proteomics workflows have been developed to capture samples consisting of small numbers of plant cells (hundreds to thousands) collected either by laser capture microdissection (LCM) or fluorescent activated cell sorting (FACS) and microfluidic nanodroplet-based sample preparation followed by nanoLC-MS/MS analysis (Balasubramanian et al. 2021).

**Fig. 3:**
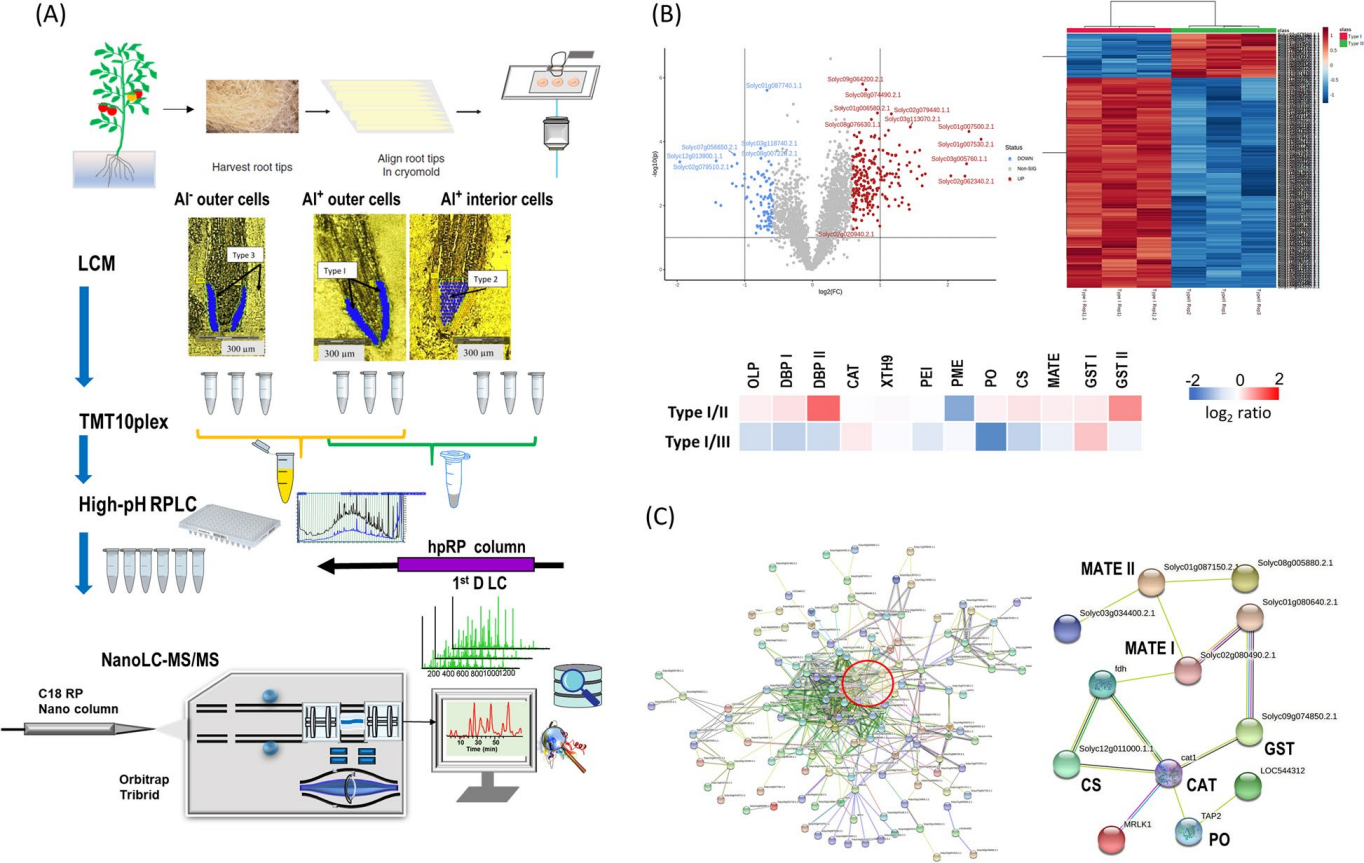
Identification of Al-induced proteomes in outer layer cells and interior of tomato root-tips. **A** TMT comparative proteomics analysis of differentially abundance proteins (DAPs) on tomato root-tips samples using laser capture microdissection (LCM); **B** Heatmap analysis based on ANOVA and Volcano plot for the DAPs between type I and type II tissues; **C** Protein network analysis by STRING (https://string-db.org/) for the DAPs found between type I and type II tissues

Studies of this type are termed single-cell-type experiments as the specimens analyzed consist of a highly reduced (but still relatively large) number of cells of a specific type (i.e., epithelial, endothelial, cortical etc.). The advantages of this approach are that it preserves the information concerning the distinct nature of the subject cell-type’s proteome. However, they do not produce high-resolution spatial information nor do they provide information concerning cell-to-cell variation. Nevertheless, this approach has proven successful in certain cases including reproductive cells (pollen grains and egg cells), mesophyll cells, and specialized epidermic cells (root hairs, guard cells and trichomes) (Dai and Chen 2012). Zhu et al. reported an application of single-cell-type proteomics using LCM in tomato seedlings grown in hydroponic tanks containing a 14.5 μM Al^3+^ solution to simulate the effects of aluminum toxicity in soil (Zhu et al. 2016b). Epidermal and cortical cells (5,000–7,000 cells per tissue type) of roots in 10-day old seedlings were collected by LCM for subsequent. protein extraction, in-gel tryptic digestion and nanoLC-MS/MS analysis. In this study, they found that a significant portion of each proteome contain proteins unique to the individual cell types, and identified several important proteins related to Al-induced morphological characteristics of roots that were not found in studies of the bulked tissue (Zhu et al. 2016b). The same groups expanded their studies to include heat-induced proteomes in meiotic pollen cells of tomato (Li et al. 2018b; Li et al. 2022a) and Al-induced proteomes of tomato root epidermal and outer cortical cells (Yang et al. 2020; Potts et al. 2022) by integrating LCM-based single-cell-type approach with TMT labeled quantitative proteomics. As shown in Fig. 3A, Al-treated outer layer cells (Type I) and interior tissues (Type II) along with control outer layer cells (Type III) in the apical meristem/cell division regions of tomato root-tips were collected by LCM for TMT10-plex quantitative proteomics analysis. Out of 6,000 quantified proteins, 313 were found to differ in abundance between the different cell types compared. These differential abundance proteins (DAPs) were used to categorize them as Al-responsive proteins (Potts et al. 2022). Figure 3B shows the volcano plot and heatmap were created between type I and type II cellular proteomes used to designate the DAPs. The complete set of DAPs identified were used to construct an association network in STRING (https://string-db.org/) using the tomato (*Solanum lycopersicum*) database (Fig. 3C). A total of 17 protein clusters and interactions were found, one of which is characterized as significant due to the increases in MATE and anti-oxidation proteins in the outer layer cells compared to those found in the interior cells (Potts et al. 2022). This data demonstrates that the single-cell-type approach is a useful strategy for the specific case of identifying novel Al tolerance mechanisms in plants, but also for the more general case of proteomics analysis of spatially resolved cells in complex tissues (Potts et al. 2022).

Recent advances in protoplasting and sequencing technologies with single-cell whole-genome amplification and single-cell RNA sequencing have allowed for direct single-cell genomic, epigenomic and transcriptomic studies in plants (Luo et al. 2020; Shaw et al. 2021). In the furtherance of the single-cell system biology strategy, this progress has stimulated efforts to develop true single-cell proteomics methods (Kelly 2020; Labib and Kelley 2020) despite the considerable technical challenges that remain, such as low sensitivity and throughput that apply to all single-cell proteomics applications, independent of phylogenetic concerns. Furthermore, plants bring additional challenges associated with the cell wall. As with all proteomic applications, the dynamic range of protein abundance can vary from a single copy to a few million copies per cell. This restricts detection to the most abundant proteins (Labib and Kelley 2020). Since the initial reports in 2018 on profiling hundreds of proteins from single mammalian cells (Budnik et al. 2018; Zhu et al. 2018b), the field has advanced steadily, albeit mostly in animal systems. Over 1,000 protein groups can now be reliably quantified by label-free analyses (Cong et al. 2020; Brunner et al. 2022) from single HeLa cells, and by isobaric labeling workflows (Dou et al. 2019; Tsai et al. 2020; Specht et al. 2021) from other mammalian cells. These successes were attributed to the recent innovations including an automated platform (nanoPOTs) for miniaturized sample preparation to reduce sample loss (Kelly 2020; Liang et al. 2021b), optimized gradients with very low flow-rates (<100 nL/min) and the introduction of next generation ion mobility MS sources which provides a >10-fold improvement sensitivity for peptides (Cong et al. 2020; Brunner et al. 2022). The development of a TMT-based multiplexed single-cell proteomics strategy incorporating a carrier proteome into one of the quantitation channels, enhances sensitivity, and improves throughput through multiplexing (Budnik et al. 2018; Dou et al. 2019). With the recent improvements allowing for the direct deposition of single cells on 96/384-well plates after cell sorting and integration of automated sample handling using the Minimal ProteOmic sample Preparation (mPOP) method, Specht et al. have reported quantifying >3,000 proteins from a single-cell proteomes using 1,490 single monocytes and macrophages in just 10-days of instrument time employing both TMT11-plex and TMT16-plex reagents for two biological replicate single-cell samples (Specht et al. 2021). It should be noted that the use of higher concentrations of carrier in TMT has been shown to compromise quantitation accuracy in the single-cell channels. To overcome this issue, optimal carrier proteome amounts and optimized MS instrument settings are required.

In addition, mass spectrometry imaging (MSI) technology has been incorporated with LCM for spatially resolved proteomics study. Compared to smaller metabolites in plant tissues, MSI for plant peptides and proteins possesses more challenges due to larger molecular weights with decreased ionization efficiency (Bjarnholt et al. 2014). For example, Cavatorta et al. demonstrated the localization of the major peach allergen, Prup3, in three different varieties of peach (Cavatorta et al. 2009) while Prup3 was present only in the outer skin of peaches that give allergic sensitivities to peach peels. Bencivenni et al. reported that non-specific lipid-transfer proteins, are one of the major human allergens in various plants that are located in tomato seeds instead of peels and pulps (Bencivenni et al. 2014). Gemperline et al. found that different distributions of endogenous peptides and protein fragments can be observed between seedlings and mature of *M. truncatula* (Gemperline et al. 2016b).

## Emerging MS based metabolomics techniques

### Sample preparation and separation technologies

#### Extraction

**Fig. 4:**
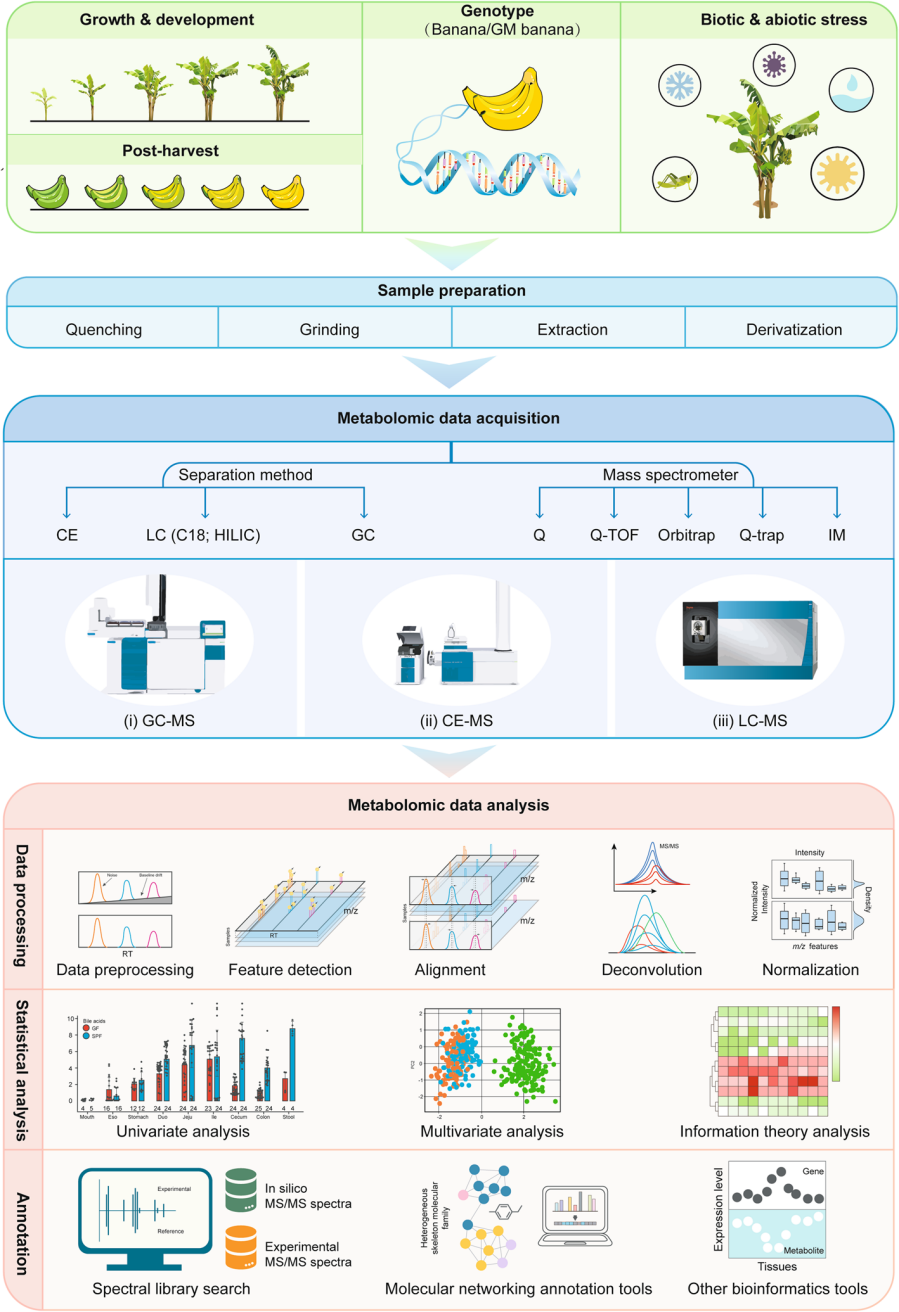
A general workflow for MS-based plant metabolomics study, including experimental design, sample preparation, metabolomic data acquisition, and metabolomic data analysis

Sample preparation is an essential step in plant metabolomics workflow (Fig. 4). An efficient extraction procedure for endogenous metabolites is the most critical step for achieving high quality plant metabolomics data. Notably, lipidomics analysis is not included in this section. The rapid turnover rate of metabolites, both primary and secondary metabolites, can occur during the extraction process (Heise et al. 2014; Rampler et al. 2021). Therefore, simple, rapid, and reproducible extraction methods are required for sample preparation. Due to the difficulties associated with extraction through cell wall, it is important to grind the plant material to a homogeneous powder before extraction. To achieve this, several strategies, such as a vibration mill (Jonsson et al. 2004), ball mill (Weckwerth et al. 2004), and Ultra Turrax (Roessner et al. 2000), have been utilized to disrupt cell walls and homogenize the sample. It is recommended that these homogenization procedures be performed in liquid nitrogen in order to avoid degradation. After homogenization, several selective metabolite extraction methods, including microwave-assisted extraction (Teo et al. 2013; Gemperline et al. 2016b; Wei et al. 2016) , ultrasound-assisted extraction (Chemat et al. 2017), high voltage electric discharge extraction (Li et al. 2019), supercritical fluid extraction (SFE) (Gallego et al. 2019), enzyme-assisted extraction (Puri et al. 2012), and solid-phase extraction (SPE) (Reyes-Garcés and Gionfriddo 2019) are commonly used in combination or sequentially. Notably, given the fact that SFE affords various advantages such as chemically stable, environmentally friendly, low toxicity, and not flammable, it has been chosen as a good extraction strategy for volatile compounds (e.g., terpenes and aromatic compounds) from plant samples (Naz et al. 2017). In recent years, a wide range of novel sorbents that are selective for the extraction of metabolites have been developed (Li et al. 2018c; Faraji et al. 2019; Rocío-Bautista and Termopoli 2019; Li et al. 2020a), such as molecularly imprinted polymers (MIPs), multiwalled carbon nanotubes (MWCNTs), metal-organic frameworks (MOFs), and covalent organic frameworks (COFs). More specifically, Li et al. prepared the cellulose magnetic molecularly imprinted polymer micro-spheres (CMMIPs) for efficient extraction and determination of plant hormone (e.g., indole-3-acetic acid) in plant tissues (Li et al. 2018d). Alireza et al. developed a MWCNT-polyaniline nanocomposite-coated platinized stainless-steel fiber for the extraction of thymol and carvacrol in medicinal plants (Ghiasvand et al. 2015). Liu et al. developed a zirconium (IV)-based MOF (UIO-67) as efficient sorbent for enrichment of eight plant growth regulators in fruit samples (Liu et al. 2016a). Recently, Li et al. reported a novel magnetic COF nanomaterial (Fe_3_O_4_@COF(TpDA)) as an adsorbent for SPE of plant growth regulators from fruits and vegetables (Li et al. 2020b). Taken together, these emerging nanostructured materials result in a reduction in sorbent amounts and higher extraction recoveries, and automation of SPE methods might facilitate the development of greener sample preparation methods. In addition, solid-phase microextraction (SPME) has been currently re-explored for metabolomics, which is a non-exhaustive extraction way particularly attractive for time-resolved or spatially metabolomics. Recently, the developments and applications of SPME as a sample preparation tool for GC-MS- and LC-MS-based metabolomics have been demonstrated and summarized (Reyes-Garcés and Gionfriddo 2019).

#### Separation method

As shown in Fig. 4, gas chromatography (GC), LC, and capillary electrophoresis (CE) are the common metabolite separation methods, which can be tandem with MS for metabolomics study. Since 2000, GC-MS has become a central platform in targeted metabolomics for volatile and nonpolar metabolites, which are major classes of primary and secondary compounds in plant science (Roessner et al. 2000). Although GC-MS has significantly advanced, two-dimensional GC (2D-GC) demonstrates superior chromatographic capabilities, including resolving power, peak capacity, and sensitivity, especially for the separation of low molecular weight (Balasubramanian et al. 2021) plant metabolites in complex samples (Tranchida et al. 2016). Recently, Gavin Sacks’ group developed solid-phase mesh-enhanced sorption from headspace (SPMESH) coupled to GC–MS for the quantitation of linalool and 3-isobutyl-2-methoxypyrazine in real grape samples (Jastrzembski and Sacks 2016). This SPMESH strategy afforded greater loading capability and was more cost-effective.

However, due to involvement of derivatization steps and other challenges in GC-MS, LC-MS has become the most valuable analytical tool for the analysis of polar and nonpolar metabolites with greater selectively and reduced ion-suppression and matrix effects. For example, Gray et al. demonstrated that a compact 1 mm i.d. column was able to reduce solvent consumption by 75% and increased sensitivity by 2-3-fold compared to the standard 2.1 mm i.d. column (Gray et al. 2015). Coelution and resistance to retention of low polarity compounds makes RPLC as a more suitable technique for separation. Thus, mobile-phase modifiers, including formic acid, ammonium formate/acetate, or ammonium hydroxide can be used (Cajka and Fiehn 2014). Tufi et al. found that the zwitterionic phase based on silica gel columns provides the optimal performance compared to four others commercial HILIC packing materials (Tufi et al. 2015). Currently, multidimensional liquid chromatography is emerging to resolve this issue, which will be summarized in the below subsection.

CE-MS has also proven to be a promising platform in metabolomics. For the separation of cations and anions, bare capillaries are typically used for CE-MS with conditions of pH < 2 and surface coated fused-silica is used when pH > 8. Tanaka et al. revealed that polymer-based fused-silica capillaries can be used to better control the EOF and lower ion adsorption (Tanaka et al. 2008). Additionally, a modification to the liquid junction in CE-MS interfaces has recently been conducted based on sheath flow or sheathless electrospray designs. Recently, a new liquid junction-based electrospray interface has been developed for automated CE-MS analysis through computer modeling of transport conditions (Krenkova et al. 2019). Using this liquid junction interface, small peptides, proteins and oligosaccharides can be well separated. Beyond that, recent decades have witnessed great improvements of CE-MS in single-cell and subcellular analyses due to its compatibility with low volume sample requirements (DeLaney et al. 2019; Kristoff et al. 2020). Williams et al. used a CE-MS method for metabolic profiling of amino acids of *Medicago truncatula* liquid suspension cell cultures in response to stress (Williams et al. 2007). Huang et al. developed an online single-cell CE-MS platform for single-cell metabolomics from a red onion (*Allium cepa*) cell, and hundreds of metabolites were successfully separated and putatively identified (Huang et al. 2021). Taken together, these results strongly suggest that CE-MS is expected to be more commonly used in single-cell metabolomics research.

In addition, super critical fluid chromatography (SFC), which utilizes liquid CO2 as a solvent, is a complementary method to GC, LC and CE. The review papers have described the advances in GC, LC, and SFC methods to improve the metabolome (Haggarty and Burgess 2017), and compared different LC and SFC strategies in terms of efficiency versus throughout, showing the performance from each method to readers (Fekete et al. 2015). With the development of new robust column and instrumentation, SFC method has been improved and applied in targeted metabolomics (West et al. 2016).

#### Combination of orthogonal chromatography techniques

Another way to reduce the coelution of metabolites and improved separation efficiency is to combine two or more separation techniques in a single analysis, such as serial combination of two different column (Alvarez-Segura et al. 2016), two-dimensional LC (2D-LC) (Stoll et al. 2007; Sandra and Sandra 2013), 2D-GC (Kouremenos et al. 2010), and 2D-SFC (Zhou et al. 2014). Among them, 2D-LC has been widely applied in untargeted metabolomics studies. An online 2D-LC can provide high-throughput and automated analysis, but short separation time might compromise the chromatographic resolution of the second LC separation (Holčapek et al. 2015). Xu’ s group recently established 2D-LC-MS and parallel column based 2D-LC (PC-2DLC)-MS approach to simultaneously perform metabolomics and lipidomics (Wang et al. 2017; Lv et al. 2020). Using orthogonal HILIC and RPLC chromatography with distinct retention mechanisms, HILIC × RPLC is capable of simultaneous separation and detection of hydrophilic and hydrophobic compounds in complex samples in a single injection, which significantly increases peak capacity and separation flux. For example, Wang et al. used stop-flow HILIC × RPLC to identify 372 lipids from 13 different classes of compounds in positive mode (Wang et al. 2013b). Zhou et al. developed a HILIC × RPLC system to separate a total of 896 peaks from *G. jasminoides Ellis (GJE)* and identify 16 active ingredients (Zhou et al. 2016b). Dang et al. demonstrated an orthogonal 2D HILIC/RPLC system for the isolation of 18 flavonoids from *S. tangutica*, which was quantitatively evaluated based on the construction of normalized 2D plots (Dang et al. 2018). Specifically, normalized 2D plots were divided into 9×10 bins, and distinct flavonoids with fractions 1–9 occupied 55 bins. Navarro-Reig and his colleagues used an HILIC × RPLC system with chemometric tools to acquire a lipidomic assessment of the effect of arsenic pollution on rice (*Oryza sativa* L.) growth (Navarro-Reig et al. 2018). Lisa et al. achieved fractionation of total lipid extracts of soya tissues into individual lipid classes using HILIC as the first separation dimension, followed by RPLC-MS separation and identification of individual species (Lísa et al. 2011).

### Advanced MS technologies for metabolomics data acquisition

#### Untargeted metabolomics

Metabolomics has rapidly grown as the major methodologies for systems biology studies, driven by new developments in MS that provides high sensitivity and high throughput coverage of the metabolome. Untargeted metabolomics analysis involves identification of global metabolites having peak intensities in a mass chromatogram processed in an unbiased way. Ideally, the MS platform instrument should provide precise mass measurements with superior mass accuracy and mass resolution for untargeted metabolomics (Treviño et al. 2015). The lack of mass resolving power will undoubtedly lead to overlap of co-eluting isobaric metabolites and will result in false positive results. To this end, untargeted metabolomics studies have been dominated by the high-resolution mass spectrometers, such as time-of-flight (TOF), Orbitrap, and Fourier transfer ion cyclotron resonance (FT-ICR) mass analyzers, which greatly improve the chemical specificity and provide high confidence in analyte identification. However, some compromises must be considered in terms of desired detection sensitivity, dynamic linearity range, and acquisition rate. Up to date, the majority of published plant metabolomics studies use Orbitrap or TOF equipment considering coverage, selectivity, and throughput (Maia et al. 2021). Recently several reviews covered well the topic of GC/LC-MS- and FT-ICR-based plant metabolomics studies (Alseekh et al. 2021; Alvarez and Naldrett 2021; Maia et al. 2021; Perez de Souza et al. 2021), so we will not cover too much in detail about the progress in this field over the past decade. In this section, we will focus on the newest technological advancements in untargeted metabolomics, such as DIA, IMS, fluxomics.

To achieve optimal analyte identification confidence, tandem MS is required to obtain characteristic fragments. Among these techniques, classic DDA remains the standard for untargeted metabolomics. However, DIA is a rapidly emerging and robust method for improved coverage of low-abundance metabolites (Tsugawa et al. 2015; Li et al. 2020c). Specifically, DIA provides more robust data than DDA acquisition technique because all fragment ions from all precursor ions are acquired simultaneously. Thus, DIA allows for increased chemical coverage of metabolites and reduced identification artifacts. One issue to note is that the molecular identification with the DIA method can be compromised due to the decoupling of precursor ions and their fragments, which can be exacerbated when large mass windows or all-fragment-ion mode are selected. Thus, these challenges promote the development of deconvolution algorithms software that matches precursor ions and fragment ions based on retention-time alignment (Tsugawa et al. 2015; Perez de Souza et al. 2021). To better distinguish metabolites of interest from background contaminants (e.g., polyethylene glycol, polypropylene glycol, siloxanes) commonly found in LC-MS/MS and enhance the coverage of metabolome, the automated intelligent workflow AcquireX (Thermo Fisher) has been developed to differentiate metabolite signals (Cho et al. 2021; Schwaiger-Haber et al. 2021). The AcquireX workflow enables which precursors to select for enhanced fragmentation (MS/MS or MS^n^) in real time by automated exclusion/inclusion list generation and updating in five consecutive LC-MS runs, allowing not only for background exclusion, but also for digging deeper into the low abundance metabolites.

For plant untargeted metabolomics, many factors still limit metabolite annotation. Ion mobility spectrometry (IMS) has been proposed for improved identification capabilities by providing an additional separation dimension within the millisecond time-window (Hofmann and Pagel 2017; Wu et al. 2021). To date, there are several available IMS platforms, such as traveling wave IMS, drift-time IMS, high field asymmetric waveform IMS, and trapped IMS, Open-loop IMS (González-Riano et al. 2020), which have been used with GC-/LC-MS for untargeted metabolomics. Given that IMS enables the CCS measurement of a wide range of metabolites and protein complexes, four-dimensional (4D) information, including retention time, CCS value, accurate molecular mass, and MS/MS fragmentation, can be obtained simultaneously. This significant increases in improving the identification confidence and prediction of unknown molecular structures. Additionally, the incorporation of IMS offers improved separation of coeluting and isobaric compounds, lower background noise, and higher mass resolution (Szykuła et al. 2019). Specifically, Jia et al. used UPLC-IMS-QTOFMS to reveal the chemical diagnostic markers for the differentiation of three Panax species (*P. ginseng*, *P. quinquefolius*, and *P. notoginseng*), which are easily misidentified and widely consumed as healthcare products (Jia et al. 2019). More than 162 compounds from fungicide-infected potato samples (*S. tuberosum*) were able to be distinguished by UPLC-IMS-QTOFMS due to its ability to produce confident compound annotations from elemental composition determination, retention times, specific CCS-values, and MS fragment spectra (Claassen et al. 2019). McCullagh et al. adopted IMS coupled to MS for profiling 6-*C* and 8-*C*-glycosylflavone isomer pairs in medicinal plants (e.g., *Passiflora *species) (McCullagh et al. 2019). Recently, Zhu’s group developed an ion mobility CCS atlas, AIICCS, to further improve annotation of both known and unknown metabolites acquired in IM-MS-based metabolomics (Zhou et al. 2020). Given the fact that CCS values are a unique physiochemical property of detected metabolites, this method will expand the chemical coverage and assessment of annotated metabolites in metabolic pathways and biological processes (Li et al. 2021a).

Fluxomics, as a new untargeted metabolomics approach to monitor the dynamic changes of metabolites in metabolic pathways, thereby provides an overview of the global regulation network with regard to transcriptional, translational, and metabolic processes (Cascante and Marin 2008). Fluxomics that combines ^13^C isotope or ^15^N labelling and computational approaches provides a deep insight into the correlation between genotype and metabolic phenotype. This technique also provides a measure of the flux through each reaction in the network, which can be considered a direct measure of the phenotype (Heux et al. 2017). Several methods have been proposed to decipher complex plant metabolic pathways. One method, metabolic flux analysis (MFA), has been widely adopted for heterotrophic tissues that lie in a metabolic and isotopic steady state (Crown and Antoniewicz 2013; Salon et al. 2017). For systems in a metabolic steady state that are isotopically dynamic, isotopically non-stationary MFA (INST-MFA) has been developed to quantify flux variations, dynamically (Wiechert and Nöh 2013; Salon et al. 2017; Wieloch 2021). Flux balance analysis (FBA) was invented as a complementary tool for deciphering genome-scale metabolic models (Shi and Schwender 2016). Recently, a machine learning-based framework has been developed to circumvent computational limitations of traditional MFA algorithms, facilitating high-throughput phenotyping and advances of synthetic biology (Wu et al. 2016; Millard et al. 2021; Wu et al. 2022). To date, fluxomics has been widely applied to determine exchange rates of a given element between various organs and nutrient use efficiency (Kichey et al. 2007) at the plant level. Specifically, fluxomics applications in the assessment of C and N use have been conducted in a range of plant species. ^15^N-based MFA methods have been applied in maize (Gallais et al. 2006), rice, wheat (Kichey et al. 2007), pea (Schiltz et al. 2005), and *B. napus* (Malagoli et al. 2005). Recently, Ma et al. and Xu et al. used ^13^C-labeling INST-MFA to estimate C fluxes in central metabolism during photosynthesis (Ma et al. 2014a; Xu et al. 2021). Cocuron et al. used isotope-labeled fluxomics to compare the metabolism of two different maize lines through ^13^C-MFA (Cocuron et al. 2019). In addition, the labeling-based fluxomics strategy has been used to determine endogenous and exogenous N and S fluxes at the whole-plant level to evaluate nutrient use efficiency (NUE) (Salon et al. 2014), as well as the N harvest index, nutrient uptake efficiency, and nutrient remobilization efficiency in *Arabidopsis* (Guiboileau et al. 2012) and maize (Li et al. 2015a) during development. MFA and INST-MFA are thus promising synergistic toolsets for uncovering the basis of C and energy conversion efficiencies in plant systems (Chen and Shachar-Hill 2012; Kruger and Ratcliffe 2021).

#### Targeted metabolomics

While untargeted metabolomics is devoted to maximizing coverage of metabolites, targeted metabolomics is concerned with the quantitative analysis or rapid profiling of a very specific set of compounds using highly selective techniques. The standardization of chromatographic conditions in GC together with reproducible spectral databases resulting from electron ionization results in a bias in favor of the use of GC-MS for targeted applications. GC-MS methods provide quantitative data for large-scale analysis of metabolites involved in central metabolism. Hence, GC-MS is the method of choice for targeted profiling and quantitation of various plant primary metabolites, with low MW, low polarity, and low boiling points. Chemical derivatization strategies are commonly required to improve ionization efficiency of non- or semi-volatile metabolites (e.g., nucleotides, fatty acids, and amino acids). Incomplete derivation not only affects the quantification of peaks, but increases the spectral complexity. Therefore, two-dimensional GC has proven to be a robust method with improved separation capacity, peak resolution, and spectral reproducibility (Almstetter et al. 2012). Targeted GC-MS methods normally adopt specific conditions for known compounds, such as select ion monitoring (SIM) and multiple reaction monitoring (MRM). Recently, Xu’s group proposed the “quasi-targeted” SIM or MRM methods instead of untargeted full-scan (Zheng et al. 2020). Yuan et al. reported a widely targeted volatilomics method based on GC-MS using MRM to profile the volatilome of rice grains (Yuan et al. 2022). To improve the quantitative capability of targeted metabolomics, use of adequate (isotope labeled) internal standards is common practice, which are ionized under nearly identical conditions as the targeted metabolites of interest (Chen et al. 2013). For targeted metabolic profiling and quantitation, triple quadrupole mass spectrometry (QqQ-MS) and quadrupole-linear ion trap mass spectrometry (QLIT-MS) are most widely used (Dettmer et al. 2007). Both SIM and MRM represent routine operation modes for QqQ-MS and QLIT-MS, which provide richer structural information, and more precise quantification analysis. Recently, a high-coverage and quantitative LC-MS/MS method using both positive and negative-mode MRM was reported for targeted 206 primary and secondary plant metabolites (Zheng et al. 2021). In past decades, LC-MS/MS for targeted metabolomics profiling and quantitation has been gaining the popularity for the broad application of plant metabolites including plant hormones such as cytokinins (Hu et al. 2021b; Pino et al. 2022), salicylic acid and glycolytic metabolites (Wang et al. 2022; Yang et al. 2022).

#### Spatially resolved metabolomics

**Fig. 5:**
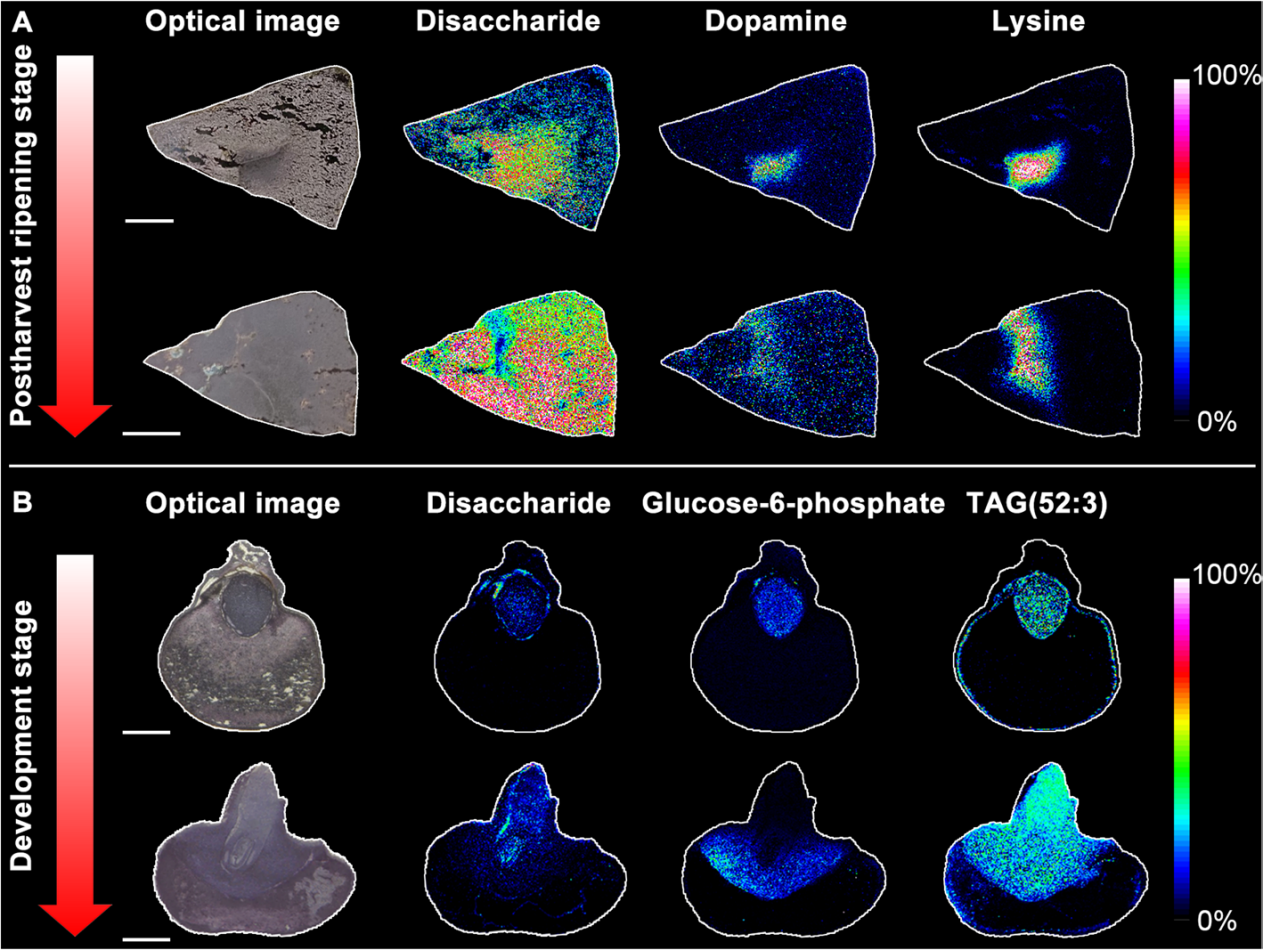
Matrix-assisted laser desorption/ionization mass spectrometry imaging (MALDI-MSI) as a molecular exploratory tool in plant science. **A** The dynamic spatial distribution of disaccharides, dopamine, and lysine within banana pulps at different postharvest ripening stage. Scale bar, 5 mm. **B** The dynamic spatial distribution of disaccharides, glucose-6-phosphate, and TAG (52:3) within maize kernels at different development stages. Scale bars, 2.5 mm

The ability to visualize metabolite distribution within plant tissues with high spatial resolution is crucial for a detailed understanding of the synthesis, accumulation, and cross-regulation of metabolites in plants (Gemperline et al. 2016a). Metabolomic analysis of bulked tissues focuses yield information concerning the mean concentrations in the bulked tissue which is of limited biological value. Thus, mass spectrometry imaging (MSI) was developed that possesses the advantages of label-free, non-specific, and visualized detection, and simultaneous analysis of hundreds of compounds in a single analysis. MSI has recently proven to be a robust technique for characterizing the spatial distributions of a wide range of metabolites within plant tissues and single cells (Yin et al. 2019; Meng et al. 2020; Li et al. 2021a; Samarah et al. 2021). Several ion sources are available for plant MSI, such as matrix-assisted laser desorption ionization (MALDI), secondary ion mass spectrometry (Wang et al. 2016), desorption electrospray ionization (Li et al. 2013), and laser ablation electrospray ionization (LAESI). The choice of ion source is dependent on metabolite species, its spatial resolution, the pressure regime, and scanning speed (Liang et al. 2016; Wang et al. 2020b). Among the available techniques, MALDI-MSI has been the most widely used technique for plant tissue imaging. As shown in Fig. 5 (unpublished data), we can observe the dynamic spatial distribution of disaccharides, dopamine, and lysine within banana pulps at different postharvest ripening stage, and the dynamic spatial distribution of disaccharides, glucose-6-phosphate, and TAG (52:3) within maize kernels at different development stages using MALDI-MSI. Sample preparation is the most crucial step for MSI to provide high-quality ion images. Improper sample preparation can damage the original sample by altering the analyte distribution, and abundance and can therefore lead to degraded detection sensitivity and spatial resolution (Dong et al. 2016b). Thus, histologic sectioning is normally adopted in MSI for roots and stems of plants (Li et al. 2016a), whereas imprinting strategies are preferred for leaves and petals due to fluctuating surfaces, wax-layer protection, and their inability to be sliced (Wu et al. 2020; Qin et al. 2021).

Recently, spatially resolved metabolomics, which integrates MSI and metabolomics methods, has been proposed for accurate determination of types, contents, and spatial differentiation of primary and secondary metabolites within plant tissues (Sumner et al. 2011; Etalo et al. 2015; Dong et al. 2016a). This integrated method has been applied in various emerging applications, such as precise localization of metabolites, biosynthesis and translocation pathways of functional metabolites, and functional gene verification. Specifically, Li et al. used MADLI-MSI to visualize the tissue-specific distribution of free flavonoids, flavonoid glycosides, and saponins, which provided deep insights into their biosynthetic pathway in legumes (Li et al. 2014). Germination and maturation are two highly active metabolic stages in seed growth and development. Bhandari et al. found that spermidine and cyclic spermidine conjugate can move from the hypocotyl to the young root of oilseed rape during seed germination, facilitating an understanding of the dynamic changes of plant metabolites at different development stages (Bhandari et al. 2015). Furthermore, organ- and tissue-specific distributions of various metabolites (e.g., flavonoid glycosides, biflavonoids, ginkgolides, and phenolic lipids) can be visualized in the root, young stem, and leaf of ginkgo (Li et al. 2018a). Dong et al. used the integrated LC-MS and MSI to visualize the steroidal glycoalkaloid (Villas-Bôas et al. 2007) pathway in wild-type and GAME25 silenced-construct tomato fruits, revealing accurate gene-metabolite relationships and novel gene-associated metabolites (Dong et al. 2020). Stopka et al. utilized an optical fiber-based laser ablation electrospray ionization mass spectrometry (f-LAESI-MS) technique to analyze individual Egeria densa leaf blade cells, revealing the metabolic differences between different cell types (Stopka et al. 2018).

However, despite its advantages, spatially resolved metabolomics has several challenges including limited detection sensitivity, spatial resolution, and molecular identification capability. Continuing efforts have been made to allow sub-micrometer resolution imaging, especially for near-field based techniques (Liang et al. 2016; Yin et al. 2019; Cheng et al. 2020). Additionally, laser post-ionization has recently been implemented in commercial MS instruments (known as MALDI-2), which significantly improves ion yields for numerous lipid classes, vitamins, and saccharides by up to two orders of magnitude (Soltwisch et al. 2015; Niehaus et al. 2019).

Taken together, spatially resolved metabolomics is still under rapid development and will become a robust strategy for plant science in near future.

### MS data analysis

The handling of MS raw data is now a mature technique for untargeted metabolomics. Several review papers have summarized the characteristics of available software for metabolomic data processing, metabolite annotation, statistics, and data interpretation, as well as the level of programing skills required to exploit their basic functions (Perez de Souza et al. 2017; Tsugawa 2018; Chaleckis et al. 2019; Misra 2021). In the subsection, we will describe the advances of MS data analysis.

#### MS data pre- or post-processing

After MS-based metabolomic data acquisition (Fig. 4), and MS data format conversion to the NetCDF, mzXML, or mzML standardized file formats, the metabolomics datasets will be processed by different computational solutions. Until now, many powerful tools have been reported to facilitate mass spectral data pre- and post-processing, such as AMDIS (Stein 1999), MetaboliteDetector (Hiller et al. 2009), MET-COFEA (Zhang et al. 2014), MetaboQC (Leonid Brodsky 2010), xMSanalyzer (Uppal et al. 2013), TracMass 2(Tengstrand et al. 2014), DecoMetDIA (Yin et al. 2019a), MET-XAlign (Lommen 2009), Normalyzer (Chawade et al. 2014), TargetSearch (Cuadros-Inostroza et al. 2009), and NOREVA (Fu et al. 2022). They have basic functions for GC-MS or LC-MS data processing, including spectral deconvolution, baseline correction, peak picking, peak annotation, alignment, and normalization (Tsugawa 2018). However, XCMS (Smith et al. 2006), Mzmine2 (Pluskal et al. 2010), OpenMS (Rost et al. 2016), and MS-DIAL (Tsugawa et al. 2015) have been widely used for different metabolomics datasets, which works based on almost similar principles. MS-DIAL, OpenSWATH (Rost et al. 2014), and MetDIA (Li et al. 2016b) are specifically used to deconvolulate the MS/MS spectra for comprehensive DIA data. Additionally, AMDIS is widely used for GC-MS based metabolomics datasets (Stein 1999). There are still several challenges in MS data processing with high false positives that are attributed to background noise, contamination, and in-source fragmentation. For example, CAMERA can perform better peak picking based on several parameters (Kuhl et al. 2012), and in-source fragmentation can be obtained both by CAMERA or RAMClust (Broeckling et al. 2014). CAMERA and other open-source software tools can cluster mass features belonging to the same metabolite, which is an important step aiming for reducing data redundancy and facilitating further statistical analyse and metabolite annotation (Broeckling et al. 2014; Uppal et al. 2017). In addition, NormAE, a deep learning model, has been developed to remove batch effects of large-scale untargeted metabolomics data (Fernández-Albert et al. 2014; Rong et al. 2020). IntCor is focused on drift removal and data normalization for LC-MS metabolomics data (Fernández-Albert et al. 2014). SPICA is built to extract relevant information from noisy datasets by analyzing ion pairs instead of individual ions (Mak et al. 2015). Several other software tools, such as MIA (Weindl et al. 2016), ICT (Jungreuthmayer et al. 2015), geoRge (Capellades et al. 2015), Massifquant (Conley et al. 2014), Allocator (Lisacek et al. 2014), Corrector (Huege et al. 2014), and X^13^CMS (Huang et al. 2014), have been developed to determine, correct, visualize and analyze mass isotope distributions in isotope labeling experiments.

#### Statistical analysis

Processed MS data, which consists of high dimensional data matrices, can be analyzed statistically by multivariate and univariate analysis. There are numerous software packages reported for statistical analyses of metabolomic data, as well as useful pipelines to facilitate statistical analysis, including MetaboLyzer (Mak et al. 2014), IPM4 (Liang et al. 2020), Pathomx (Fitzpatrick et al., 2014), MetabR (Ernest et al. 2012), COVAIN (Sun and Weckwerth 2012), metaP-server (Kastenmüller et al. 2021), OpenMS (Rost et al. 2016), MathDAMP (Baran et al. 2006) , RepExplore (Glaab and Schneider 2015), Metabomxtr (Nodzenski et al. 2014). Recently, other more specialized tools, including LipidSuite (Mohamed and Hill 2021), MSEA (Xia and Wishart 2010), MPA-RF (Huang et al. 2013), and probabilistic principal component analysis (PPCA) have been developed (Nyamundanda et al. 2010). Specifically, LipidSuite offers a step-by-step workflow for MS data processing, differential analysis and enrichment analysis of lipidomics data (Mohamed and Hill 2021). MSEA offers three different enrichment analyses for metabolomic data (Xia and Wishart 2010). PPCA addresses some of the limitations of PCA, and probabilistic principal component and covariates analysis provides a flexible approach to jointly model metabolomic data and additional covariate information (Nyamundanda et al. 2010). MPA-RF helps to identify the informative biomarkers in complex metabolic datasets (Huang et al. 2013). Subpathway-GM is a method for pathway analysis that integrates information from genes and metabolites, as well as their positions and cascade regions within the given pathway (Li et al. 2013).

#### Metabolite annotation

Accurate metabolite annotation is very vital for data interpretation. But it is still considered as the most challenging step for MS-based metabolomics study, which largely depends on retention time, accurate mass, mass spectra, MS/MS fragmentation pattern, and other sample-related information. The guide for metabolite annotation by current MS-based cheminformatics is reviewed by Tsugawa et al. (Tsugawa 2018). As a guideline for annotation, the Metabolomics Standards Initiative has recommended four confidence levels: from level 1 to level 4 (Sumner et al. 2007), and metabolites identified using authentic standards compounds are considered as level 1. Once a metabolite is identified, its MS/MS spectra can be deposited in databases. Therefore, it is important to establish a spectral database covering accurate mass with retention time using authentic standards compounds for leveraging metabolite annotation, although generation of spectral library will be expensive with the high cost of commercial standard compounds. MS/MS spectra are currently the starting point for most of MS-based metabolite annotation approaches to search through different experimentally derived databases, including experimentally derived databases, including NIST, METLIN, MoNA, MassBank, European MassBank, mzCloud, GMD, GNPS, ReSpect and LipidMaps, which are covered in other comprehensive review papers about a complete databases (Vinaixa et al. 2016; Perez de Souza et al. 2017; Blazenovic et al. 2018). Some in silico generated MS/MS spectra are also included in popular compound databases for metabolite annotation, such as PubChem, Chemspider, HMDB, KEGG, ChEBI, ChemBank, and universal natural product database (UNPD) (Wang et al. 2016; Blazenovic et al. 2018). Accordingly, MetaboSearch (Fernandez-Fuentes et al. 2012), PUTMEDID-LCMS (Brown et al. 2011), MFSearcher (Sakurai et al. 2013), MetAssign (Daly et al. 2014), MS-FINDER (Tsugawa et al. 2016), HR3 (Lommen 2014), SIRIUS (Böcker et al. 2009), and MS2Analyzer (Ma et al. 2014b), are developed for interrogating and matching unannotated mass spectra from those databases. Recently, METLIN, as the largest individual collection of MS/MS experimental data acquired in multiple collision energies, has been directly integrated with XCMS online processing platform (Guijas et al. 2018b).

One of the major obstacles for proper MS-based metabolite annotation is the limited number of characterized authentic standards for the majority of the different compounds derived from plants. Thus, several alternatives have been developed to support metabolite annotation, some large databases expand the coverage by including in silico generated MS/MS spectra of known compounds based on machine learning or quantum mechanics calculations. MetFrag (Ruttkies et al. 2016), MolFind (Menikarachchi et al. 2012), CFM-ID (Allen et al. 2014), MAGMa (Ridder et al. 2014), CSI: FingerID (Dührkop et al. 2015), MetFusion (Gerlich and Neumann 2013), and MetFrag (Ruttkies et al. 2016), can be used to predict molecular properties of unknown compounds by their tandem mass spectra based on the integration of machine learning model and known mass spectra. CANOPUS uses a deep neural network to predict 2,497 compound classes from fragmentation spectra, including all biologically relevant classes (Dührkop et al. 2020). DLEMMA is a valuable tool and is analogous to DNA arrays in that it enables the identification and relative quantification of differential metabolites in a single sample (Feldberg et al. 2009). ChemDistiller combines automated large-scale annotation of metabolites using tandem MS data with a compiled database containing tens of millions of compounds with pre-calculated ‘fingerprints’ and fragmentation patterns (Laponogov et al. 2018). One issue to note is that, molecular networking has been widely used in the last half decade, which relies on the idea that structurally related compounds have similar MS/MS fragmentation, and thus generate spectra similarity networks (Yang et al. 2013b). The GNPS provides an open-source platform for sharing mass spectral data and data analysis based on molecular networking (Wang et al. 2016). As shown in Table 2, we summarized the function characterization of the advanced molecular networking tools for metabolite annotation of LC-MS data, such as IIMN (Schmid et al. 2021), NetID (Chen et al. 2021), FBMN (Nothias et al. 2020), SIRIUS 4 (Duhrkop et al. 2019), NAP (da Silva et al. 2018), MSHub/GNPS (Aksenov et al. 2021), MetDNA (Shen et al. 2019), Metwork, MolNetEnhancer (Ernst et al. 2019), Qemistree (Tripathi et al. 2021), DEREPLICATOR+ (Mohimani et al. 2018), DEREPLICATOR (Mohimani et al. 2017), ISDB (Allard et al. 2016), MS2LDA (van Der Hooft et al. 2016) etc. To date, the strategy based on molecular networking has shown outstanding potential to increase the number of signals assigned to a putative chemical structure in MS based metabolomics, which has been comprehensively reviewed (Fox Ramos et al. 2019; Perez De Souza et al. 2020; Beniddir et al. 2021).

**Table 2:** Advanced molecular networking tools for metabolite annotation of LC-MS/MS data

Tool name	Function characterization	Reference
IIMN	A tool integrates chromatographic peak shape correlation analysis into molecular networks to connect and collapse different ion species of the same molecule.	(Schmid et al. 2021)
NetID	An algorithm optimizes a network of mass spectrometry peak connections based on MS1 mass differences corresponding to the gain or loss of relevant chemical moieties, and MS2 spectral similarity.	(Chen et al. 2021)
Qemistree	A tree-based approach for computing and representing chemical features from tandem MS-based metabolomics studies, which is based on the hierarchical organization of molecular fingerprints predicted from MS/MS fragmentation spectra.	(Tripathi et al. 2021)
FBMN	A method that bridges popular MS data processing tools for LC-MS/MS and molecular networking analysis on GNPS, enabling the characterization of isomers, incorporation of relative quantification, and integration of ion mobility data.	(Nothias et al. 2020)
CliqueMS	A computational tool that annotates redundant MS1 features by constructing a similarity network between coelution profiles and a calculated natural frequency of adduct formation observed in real complex biological samples and pure compounds, which produces accurate annotations for a single MS1 spectrum.	(Senan et al. 2019)
MetWork	An annotation propagation tool, which based on MS2 data, organized in molecular network, a collaborative library of reactions, and a MS2 spectra prediction module.	(Beauxis et al. 2019)
MetDNA	A metabolic reaction network-based recursive algorithm that characterizes initial seed metabolites with MS2 spectra, and utilizes their experimental MS2 spectra as surrogate spectra to annotate their reaction-paired neighbor metabolites.	(Shen et al. 2019)
SIRIUS 4	A tool integrates high-resolution isotope pattern analysis and fragmentation trees to provide an assessment of molecular structures from MS2 data for large datasets and propagation of annotation through molecular networks.	(Duhrkop et al. 2019)
MolNetEnhancer	A software package that unites the output of several tools, including mass spectral molecular networking, unsupervised substructure discovery, and in silico structure annotation to illuminate structural details for each fragmentation spectrum.	(Ernst et al. 2019)
DEREPLICATOR +	A tool for search the entire GNPS and identifies variants of known metabolites using molecular networking, which improves the identification of peptidic natural products, polyketides, terpenes, benzenoids, alkaloids, flavonoids, etc.	(Mohimani et al. 2018)
NAP	An on-line tool that uses a combination of molecular networks, based on spectral similarity, together with in silico fragmentation, to enable the scientific community to strengthen their MS annotations.	(da Silva et al. 2018)
DEREPLICATOR	A new dereplication algorithm that searches MS/MS spectral datasets against the database of peptidic natural products (PNPs), which enables high-throughput PNPs identification based on molecular networking.	(Mohimani et al. 2017)
MS2LDA	An unsupervised method that extracts common patterns of mass fragments and neutral losses-Mass2Motifs from the collection of fragmentation spectra, which can be used to annotate molecules.	(van der Hooft et al. 2016)
ISDB	An innovative dereplication strategy based on the combination of molecular networking with an extensive in-silico MS2 fragmentation database of natural products.	(Allard et al. 2016)
GNPS	An open-access knowledge base for community-wide organization and sharing of raw, processed or identified MS2 spectrometry data.	(Wang et al. 2016)

## Metabolomics applications to plant research

### Gene function characterization and metabolic pathway exploration

Plant endogenous metabolites are indispensable for human being and the plant itself. Hence, comparative metabolomics has been very powerful to highlight the metabolic differences in different plant species (Wang et al. 2014a) and in the cross-comparison of metabolite quantitative trait loci (mQTL) in populations of the same plant species (Wen et al. 2014). Metabolomics also allows the determination of metabolites in plants with vital roles in tolerance to abiotic or resistance to biotic stress. Integrating metabolomics with other-omics technologies is used to pinpoint the causal genes and further exploration of metabolic pathway, which becomes more and more important for marker-assisted breeding and metabolic engineering to target key pathways of plants. Previous studies show that multi-omics analysis using near isogenic lines and natural variants of a given plant could be used to identify new metabolites (Tsugawa et al. 2021).

Firstly, most traits of crops are polygenic, therefore, their genetic basis can be readily elucidated with the strategies of quantitative genetics. With the rapid development of metabolomics- and genomics-related technologies, mQTL and metabolite genome-wide association studies (mGWAS) have been commonly used to dissect the genetic architectures underlying the varied metabolites in plants in the last 15 years, which will generate the linkages or associations between chromosomal locations and metabolite contents. Subsequently, the candidate genes included within the genomic interval or adjacent to the associated marker loci are identified in many plant species with annotated genomes, which have been comprehensively reviewed (Fang and Luo 2019). Recently, by integrating comparative metabolomics and mGWAS analyse, Liang et al. identify 10 candidate genes significantly associated with the abundances of 37 metabolites, which are biomarkers related to salt stress tolerance (Liang et al. 2021a). Chen et al. identify 26 candidate genes and validate two genes involved in the flavonoid decoration pathway of wheat kernel through wheat mGWAS study (Chen et al. 2020a). And numerous studies have been reported to characterize genes and dissect the genetics of metabolic traits in other crops using a similar multi-omics integration approach, such as in cucumber (Shang et al. 2014), in maize (Wen et al. 2014; Wen et al. 2016), in tomato (Sauvage et al. 2014; Alseekh et al. 2015; Zhu et al. 2018a), and in rice (Chen et al. 2014; Chen et al. 2016). We also conducted a study on integrated metabolomics, genomics, and other technologies to validate the function of several candidate genes in rice (Liu et al. 2020).

Secondly, the strategy of integrating metabolomics with transcriptomics also help to unveil the relationship between the genotype and phenotype of an organism and identify the function of core genes involved in specific metabolic pathways, such as proanthocyanidin biosynthesis in *Arabidopsis thaliana* (Kitamura et al. 2010), noscapine (Winzer et al. 2012), tanshione biosynthesis in *Salvia miltiorrhiza* (Gao et al. 2014), flavonoids and amino acids biosynthesis in *Camellia sinensis* L. (Huang et al. 2018), anthocyanin biosynthesis in *Triticum aestivum* L. (Wang et al. 2021b). For example, we have integrated metabolomics with transcriptomics to demonstrate the carbon metabolism and plant hormones regulation in *Vigna radiata* during post-germination seedling growth (Wang et al. 2020a). Many previous studies used the same multi-omics strategy to reveal the metabolic shifts or metabolic regulation occurred during plant development (Rohrmann et al. 2011; Asfaw Degu et al. 2014), or to understand the global metabolic responses to different kinds of stresses (Hirai et al. 2004; Oates et al. 2015; Agarrwal et al. 2016).

Thirdly, the strategy of integrating metabolomics with proteomics has been also used to demonstrate the alterations in the metabolic and protein composition of a cell required to manifest phenotypic plasticity by plants, such as responses to perturbation of glucosinolate biosynthesis (Chen et al. 2012), and to different stress (Kushalappa and Gunnaiah 2013). Amiour et al. have integrated metabolomics, transcriptomics, and proteomics to identify key steps involved in nitrogen metabolism in maize (Amiour et al., 2012). Recently, we have conducted comparative metabolomics, proteomics, and transcriptomics analyses between the winter and spring tender shoots of a novel ever-growing tea tree, and observed phytohormone, amino acid and energy metabolism response to winter adaptation (Dai et al. 2021). By integrating metabolomics with proteomics datasets for banana carotenoid study, we found that increased abundance of carotenogenesis-associated proteins alongside elevated carbohydrate accumulation contribute to high carotenoid content in banana pulp during its development, implying that a multi-target approach is necessary in order to improve carotenoid content in banana (Heng et al. 2019).

### Safety assessment of genetic modified (GM) plants

Metabolic engineering is the use of particular metabolites for plant improvement strategy, that involves the transgenic expression and RNAi silencing of targeted genes encoding enzymes involved in the specific metabolic pathway to modulate the metabolite biosynthesis. However, beyond the intended changes, there are certain unintended changes that occur in these plants. Therefore, untargeted metabolomics technology has long been suggested and applied for safety assessment of the GM plants (Cellini et al. 2004; Heinemann et al. 2011; Stewart and Shepherd 2013; Guijas et al. 2018b). Metabolic profiling of transgenic rice, which has a mutated anthranilate synthase gene for feedback inhibition-insensitive synthesis of tryptophan, reveal the elevated levels of free tryptophan and only minor changes in levels of other free amino acids (Wakasa et al. 2006). Several other crops such as soy (Padgette et al. 1996), potatoes (Hellwege et al. 2000), wheat (Obert et al. 2004), and alfalfa (McCann et al. 2006) have also been analyzed at the metabolic level to establish substantial equivalence between their GM and non-GM counterparts. Knock-out mutants of the phenylalanine ammonia lyase involved in the phenylpropanoid pathway in *Arabidopsis* accumulate higher levels of phenylalanine and also show perturbed metabolisms of other aromatic amino acids (Rohde et al. 2004). Recently, the unintended metabolic consequences of the BAR gene, which encodes a bacterial acetyltransferase that has N-acetylation activity towards the herbicidal amino acid phosphinothricin, expressed in transgenic crops is another illustration of the utility of untargeted metabolomics in assessing new genetically engineered traits in crops (Christ et al. 2017). Until now, numerous studies have illustrated the utility of untargeted metabolomics to improve GM crop safety assessment (Catchpole et al. 2005; Shepherd et al. 2015).

### Natural products chemistry

Plant natural products (NPs) represent a large family of diverse chemical entities with a wide variety of biological activities that have been found with multiple uses, notably in human and veterinary medicine and in agriculture (Katz and Baltz 2016). For NP discovery, the separation of metabolites is usually performed using GC or HPLC columns, and the eluants are usually analyzed directly by MS detection. Several MS-based databases and software tools are now applied for NPs identification, such as ReSpect (Sawada et al. 2012) and GNPS (Wang et al. 2016). Elucidation of the metabolic pathways of NPs would help to determine their efficacy and safety. Integrating metabolomics and next-generation sequencing data help to elucidate the pathways of NP metabolism in medicinal plants. For example, the metabolic pathways of benzoisoquinoline and monoterpenoid indole alkaloids, cannabinoids, caffeine, ginsenosides, with anolides, artemisinin, and taxol are elucidated (Scossa et al. 2018). Furthermore, the labelling approach in metabolomics such as ^13^C-based metabolomics studies, can also be applied for discovering novel secondary metabolism (Creek et al. 2012; Ellis and Goodacre 2012).

Using metabolomics technology, the metabolic maps of many bioactive NPs have been reported, such as myrislignan (Yang et al. 2017), triptonide (Hu et al. 2018), osthole (Zhao et al. 2018), dehydrodiisoeugenol (Lv et al. 2017) and so on. Biological activities of NPs can also be evaluated and their pharmacological effects can be predicted by MS-based metabolomics, which has been extensively reviewed (Zhao et al. 2018). Furthermore, metabolomics has also been employed in the quality control of NPs, being used to monitor the variation of metabolic profiles among individuals, environmental alterations during growth and harvesting, post harvesting treatment, extraction, and method of isolation (Salem et al. 2020). Metabolomics, combined with unsupervised principal component analysis and supervised partial least square analysis/partial least square analysis with discriminant analysis, are the common methods used in the quality control. For example, Wang et al. used this method to study the effect of location on the percentage of various constituents of chamomile (*Matricaria recutita* L.) (Wang et al. 2004).

## Challenges and future perspectives

Over the past decade, technological advances have led to significant improvements for in-depth proteomics and metabolomics analyses and have enabled the development of true single-cell proteomics and metabolomics technologies. However, despite all the progress, significant technical challenges remain mainly due to the inherent nature of the complex molecules involved. This is particularly true for single-cell proteomics and metabolomics given the limited amounts of materials available. Foremost among these challenges is sample preparation. To maximize proteome or metabolome coverage, one must develop an efficient and unbiased solubilization method for all cellular proteins and metabolites. When dealing with plant tissues, the cell wall poses an additional challenge in preparation of cells for FACS from plant tissue. In order to render the cells mobile, plant cells will have to be reduced to protoplasts by the removal cell wall through enzymatic degradation. This is a slow process that can, by itself, alter the composition of the cell’s proteome creating a highly artificial sample. A sufficient number of controls must be included to demonstrate that the proteomics information obtained after this process is at all relevant to biological question being asked and not just a consequence of the stress introduced by the removal of the cell wall.

Continuous developments concerning the technical aspects of high throughput sampling with miniaturization, fast and high resolution/accuracy MS with superior sensitivity, and machine learning-based data analysis are expected to push the boundaries enabling new capabilities allowing for single-cell measurement of PTMs, and improved dynamic range ultimately leading towards full coverage of both proteomes and metabolomes with quantitative accuracy. These front-end sample processing developments have propelled MS to become the central analytical tool. An integration of post-ionization gas-phase fractionation with advanced MS instruments is seeing increased use as an additional and complementary orthogonal separation to column chromatography. This development is of particular benefit to single-cell proteomics (Clark et al. 2021; Brunner et al. 2022). Therefore, the use of IMS is expected to expand particularly for single cell proteomics and metabolomics in future.

Further development of MSI in both proteomics and metabolomics is highly anticipated. In recent years, MSI is becoming more widely used for plant-omics as it provides molecular analysis of tissue with the spatial distribution of the different analytes in the tissue sample. To date, MSI has been the most impactful tool for metabolomic applications including single cell metabolomics. One advantage of MSI-MALDI is that matrix application and ionization are conducted directly on tissue, which is superior to cell isolation for metabolome integrity while retaining the relative localization of cells and allowing for assessment of the intercellular space (Korte et al. 2015). However, there are many challenges for MSI analysis that include data analysis, reproducibility, low throughput, poor quantitative accuracy and low ionization efficiency for plant peptides and proteins. Nevertheless, it is anticipated that further refinement and development of MSI platforms and associated data analysis software are necessary for reliable in situ analysis of proteins/peptides and metabolites reflecting specific plant phenotypes.

Many effective and intelligent MS analysis strategies with real-time instrument control and decision making have emerged as a means for improving the quality of acquired spectra and maximizing confident identification and quantitative accuracy. A recently developed RTS MS3 acquisition method on the Orbitrap Tribrid MS instrument is a good example for enhanced TMT-based quantitative proteomics (Erickson et al. 2019). Another is the use of the AcquireX intelligent data acquisition workflow for in-depth MS^n^ analysis of numerous low abundance metabolites in untargeted metabolomics.

Interpreting the tens of thousands of MS/MS spectra, including chimeric spectra from multiple co-isolated peptides, and translating them into biological information is another hurdle. Automated MS/MS-based peptide identifications and metabolite annotations have relied on database search engines with statistically based scoring algorithms. Thus, we expect that improved database search engines, particularly deep learning-based algorithms will be developed for enhanced search outcome. In untargeted metabolomics and DIA-based proteomics, spectral library databases are required for confident identities (Tada et al. 2019; Zhang et al. 2020a). However, building these reference libraries from chemical standards or DDA of peptide fractions is limited in size relative to known chemicals or initial DDA identities. Recently, development of algorithms predicting in silico MS/MS spectra has shown great promise (McEachran et al. 2019; Chao et al. 2020) for DIA in-depth proteome (Hu et al. 2016). Thus, we anticipate the current search engines and algorithms of in silico MS/MS spectra annotation will be further improved.

Perhaps the biggest challenge for future proteomics and metabolomics is to interrogate the acquired data with multi-omics datasets in system biology studies. We have reached a stage where high-throughput sequencing and MS cover all the large-scale disciplines we can define: genomics, transcriptomics, proteomics, metabolomics, and lipidomics. With this amount of information, a systematic multi-omics integration (MOI) of the large data sets from all those techniques remains a need (Gomez-Cabrero et al. 2014). Despite the unique challenges in plants, three levels of MOI were summarized covering element-based, pathway-based and mathematical-based integration (Jamil et al. 2020). PaintOmics, a web server for the integrated analysis and visualization of multiple omic data has been continually developed in the past decade, allowing researchers for interactive exploration of their multi-omics datasets including transcriptomics, proteomics and metabolomics (Hernandez-de-Diego et al. 2018; Liu et al. 2022). Since machine learning has been demonstrated to aid in integrating the multi-omics platforms for plant-environment interaction (Moore et al. 2019) and precision breeding (Weckwerth et al. 2020), we anticipate the development of effective machine learning algorithms will be one of the focuses in facilitating plant MOI research.

## Data Availability

All data discussed in this review are associated with the supporting primary research papers except of Fig. 5 (unpublished data) which will be available upon request.

## Abbreviations

AP-MS: Affinity purification coupled to mass spectrometry
CE-MS: Capillary electrophoresis tandem mass spectrometry
ChEBI: Chemical entities of biological interest
Co-IP: Co-immunoprecipitation
CCS: Collision cross-section
CID: Collision induced dissociation
COFs: Covalent organic frameworks
DDA: Data dependent acquisition
DIA: Data independent acquisition
DAPs: Differential abundance proteins
ECD: Electron capture dissociation
ETD: Electron transfer dissociation
EOF: Electroosmotic flow
FDR: False discovery rate
FACS: Fluorescent activated cell sorting
FT-ICR: Fourier transfer ion cyclotron resonance
FWHM: Full width at half maximum
GC-MS: Gas chromatography tandem mass spectrometry
GO: Gene Ontology
GM: Genetic modified
GNPS: Global natural products social molecular networking resource
GMD: Golm metabolome database
HS-SPME: Headspace-solid-phase microextraction
HCD: Higher energy collisional dissociation
HMDB: Human metabolome database
HILIC: Hydrophilic interaction chromatography
IMAC: Immobilized metal affinity chromatography
IMS: Ion mobility spectrometry
iTRAQ: Isobaric tags for relative and absolute quantification
INST-MFA: Isotopically non-stationary metabolic flux analysis
KEGG: Kyoto Encyclopedia of Genes and Genomes
LFQ: Label free quantitation
LCM: Laser capture microdissection
LCM: Laser capture microdissection
LC-MS: Liquid chromatography tandem mass spectrometry
MSI: Mass spectrometry imaging
MoNA: Massbank of north america
MALDI: Matrix-assisted laser desorption ionization
MFA: Metabolic flux analysis
mGWAS: Metabolite genome-wide association studies
METLIN: Metabolite link
mQTL: Metabolite quantitative trait loci
MOFs: Metal-organic frameworks
mPOP: Minimal ProteOmic sample Preparation
MIPs: Molecularly imprinted polymers
MOI: Multi-omics integration
MRM: Multiple reaction monitoring
MWCNTs: Multiwalled carbon nanotubes
nanoPOTs: Nanodroplet processing in one pot for trace samples
NIST: National institute of standards and technology
NPs: Natural products
PTMs: Post translational modifications
PPIs: Protein–protein interactions
QLIT-MS: Quadrupole-linear ion trap mass spectrometry
RTS: Real-time search
RPLC: Reversed phase liquid chromatography
ReSpect: RIKEN MSn spectral database for phytochemicals
SIM: Select ion monitoring
SP3: Single-pot solid-phase-enhanced sample preparation
SPE: Solid-phase extraction
SPMESH: Solid-phase mesh-enhanced sorption from headspace
SPME: Solid-phase microextraction
SILAC: Stable-isotope labelling by amino acids in cell culture
SCX: Strong cation exchange
SFC: Super critical fluid chromatography
SPS: Synchronous precursor selection
TMT: Tandem mass tag
TOR: Target of rapamycin
TPP: Thermal proteome profiling
TiO_2_: Titanium dioxide
QqQ-MS: Triple quadrupole mass spectrometry
2D-LC: Two-dimensional liquid chromatography
UPLC: Ultra performance liquid chromatography
UVPD: Ultraviolet photodissociation
UNPD: Universal natural product database
YTH: Yeast two hybrid
ZrO_2_: Zirconium dioxide
